# Magnitude and Moderators of Depressive Symptom Improvement in Digital Placebo Arms: Systematic Review and Meta-Analysis

**DOI:** 10.2196/100426

**Published:** 2026-10-09

**Authors:** Takashi Hosono, Rinka Tsutsumi, Yuki Niwa, Masuo Kondoh

**Affiliations:** 1 Graduate School of Pharmaceutical Sciences The University of Osaka Suita, Osaka Japan; 2 Clinical Research, R&D NS Pharma, Inc Paramus, NJ United States; 3 Faculty of Pharmaceutical Sciences The University of Osaka Suita, Osaka Japan; 4 Center for Infectious Disease Education and Research (CiDER) The University of Osaka Suita, Osaka Japan; 5 Global Center for Medical Engineering and Informatics The University of Osaka Suita, Osaka Japan

**Keywords:** digital health, digital intervention, digital therapeutics, DTx, randomized controlled trial, RCT, psychiatry, psychiatric, depression, depressive disorders

## Abstract

**Background:**

Depressive disorders are one of the most prevalent psychiatric disorders globally and impose considerable individual and societal burdens. Psychotherapy, including cognitive behavioral therapy, is recommended as a first-line treatment especially for mild to moderate depressive disorders. However, face-to-face psychotherapy is often limited by issues of accessibility and cost. Digital therapeutics (DTx)—defined as health software intended to treat or alleviate a disease, disorder, condition, or injury by generating and delivering a medical intervention that has a demonstrable positive therapeutic impact on a patient by the International Organization for Standardization—have gained increasing attention as alternatives for overcoming these hurdles. With advances in digital technology, digital placebos have begun to be used as comparators in the clinical trials of DTx to assess the effects of active interventions accurately. However, the characteristics of clinical trials, the magnitude of digital placebos effects, and their moderators remain poorly understood.

**Objective:**

This study aimed to characterize clinical trials using digital placebos in control arms and to assess the magnitude and moderators of Patient Health Questionnaire-9 (PHQ-9) improvement in digital placebo arms.

**Methods:**

The blinded randomized controlled trials (RCTs) evaluating PHQ-9 by setting digital placebos as comparators were identified by searching MEDLINE, Scopus, Web of Science, PsycINFO, CINAHL, Cochrane Central Register of Controlled Trials, ClinicalTrials.gov, and ISRCTN in November 2025. The characteristics of the RCTs and of the digital placebos were reviewed systematically. The meta-analysis including subgroup analyses and meta-regressions were conducted to investigate the magnitude and the moderators of the PHQ-9 improvement in digital placebo arms.

**Results:**

A total of 29 papers and 30 trials with 5680 participants were included in this systematic review and meta-analysis. The most common trial design was 2-arm, parallel-group RCTs conducted in a single country, adopting “Replaced” and “Mobile” as the placebo approach and delivery type, respectively. The pooled effect size for all the included trials, representing PHQ-9 changes within control arms, was Hedges *g*=0.44 (95% CI 0.29-0.59) with high heterogeneity (*I*^2^=93.2%, τ²=0.14). Subgroup analyses and univariable regression showed that “Primary psychiatric disorder” group (*P*=.008) and baseline PHQ-9 score (*P*<.001) were the independent moderators of the improvement in digital placebo arms. Multivariable regression indicated that these 2 variables are the major contributing factors of the high heterogeneity (*I*^2^=82.1%, τ²=0.07, *R*^2^=51.5%).

**Conclusions:**

Our findings indicate that improvement in digital placebo arms is not negligible and should be incorporated into trial design. The factors identified in this study will be useful when estimating expected improvement in control arms and planning adequately powered future DTx clinical trials, alongside other relevant conceptual and methodological factors that may influence digital placebo effects.

**Trial Registration:**

PROSPERO CRD420251118826; https://www.crd.york.ac.uk/PROSPERO/view/CRD420251118826

## Introduction

Depressive disorders are one of the most prevalent psychiatric disorders, affecting 280 million people including 23 million children and adolescents in the world in 2019 [1]. The COVID-19 pandemic triggered 25% increase of anxiety and depressive disorders worldwide [2]. A large survey showed that overall prevalence of depressive disorders after the pandemic remains higher than prepandemic levels [3].

Psychotherapy such as cognitive behavioral therapy is recommended as one of the first-line treatment options especially for mild to moderate depressive disorders, and as an adjunct to pharmacotherapy for moderate to severe cases [4-6]. However, face-to-face psychotherapy is often limited by issues of accessibility and cost. Alternatively, digital interventions including digital therapeutics (DTx) have gained increasing attention as alternatives for overcoming these hurdles [7].

DTx is defined by the International Organization for Standardization as health software intended to treat or alleviate a disease, disorder, condition, or injury by generating and delivering a medical intervention that has a demonstrable positive therapeutic impact on a patient [8]. The number of clinical trials of DTx conducted in the world has also increased, largely driven by psychiatric research [9]. Traditionally, no intervention, waiting list, or treatment-as-usual was widely used as the comparator in the DTx clinical trials mainly because maintaining blinding with appropriate comparators was technically challenging [10]. With advances in digital technology, digital placebos have begun to be used as comparators in the DTx clinical trials to assess the true effects of active interventions in a blind manner [11].

However, there is no globally accepted definition of digital placebos, and the digital placebo conditions used in DTx clinical trials are conceptually and methodologically more heterogeneous than drug placebos in pharmacotherapy trials [12]. Because of this complexity, the digital placebo effect is considered as collection of various effects and processes, and careful study design is required to investigate the potential of active interventions [13]. Consequently, the magnitude of overall digital placebo effects and their moderators have not been fully investigated, and the limited understanding makes it difficult to assess the efficacy of DTx in clinical trials appropriately [12]. Our previous study showed a statistically significant, small-to-moderate improvement in digital placebo arms on generalized anxiety disorder (GAD) symptoms and identified target population, placebo approach, and baseline GAD scores as moderators of the improvement. However, only 3 GAD symptom scores, which were the most frequently reported measures, were included in the meta-analysis to control heterogeneity. Further studies on other diseases were also needed to investigate the generalizability of the findings [14].

In this systematic review and meta-analysis, we aimed to review the characteristics of the blinded randomized controlled trials (RCTs) that used digital placebos as comparators and Patient Health Questionnaire-9 (PHQ-9) as the assessment score systematically, and to investigate the magnitude of improvement in digital placebo arms and their moderators on depressive symptom scores in meta-analysis. Although PHQ‑9 is a self‑reported screening measure completed by individuals themselves rather than health care professionals, it is widely used as a reliable and valid instrument for assessing the severity of depressive symptoms widely too [15,16]. To our knowledge, this is the first study that rigorously assessed the magnitude and the moderators of depressive symptom improvement in digital placebo arms.

## Methods

This systematic review and meta-analysis followed the PRISMA (Preferred Reporting Items for Systematic Reviews and Meta-Analyses) reporting guideline (Multimedia Appendix 1) [17], and the protocol was registered in PROSPERO (International Prospective Register of Systematic Reviews) (CRD420251118826).

### Search Strategy

With respect to PICO (Population, Intervention, Comparison, Outcome), each trial was required to include adults aged 18 years or older but was not limited to diagnosed patients with depressive disorders to generate a broader data pool. Instead, the impact of different target populations was investigated in subgroup analyses. Interventions were limited to active DTx, but software-based interventions that did not strictly meet the International Organization for Standardization definition were also included if they intended to influence symptoms (eg, prevention-focused programs without established therapeutic impact). Comparisons were limited to digital placebos and further details are provided in the next paragraph. Only PHQ-9 was adopted as depressive symptom score to control heterogeneity, but any mode of PHQ-9 administration (eg, digital self-report, paper-based completion, clinician-assisted administration, or independent self-completion) was accepted in this study [18].

Without a globally unified definition of digital placebos, we defined them based on previous research as “comparators designed to mimic the DTx (eg, with a similar design, components, and duration of treatment as the DTx), but the DTx active principle or component being removed or reduced in intensity” [19]. A control with a different delivery type from the intervention was not regarded as the digital placebo in this study. For example, delivering control via web while providing the intervention through virtual reality was not considered as the digital placebo. Treatment or training delivered only through online communication such as telemedicine was not regarded as digital placebos either. The trials with passive control conditions in which participants were provided only with access rights to information were also excluded because these conditions were not considered to mimic the DTx intervention. Noninferiority and equivalence trials against comparators were regarded as active-versus-active designs and were excluded from this study. When a trial included more than 1 digital placebo arm, we selected the arm that was most consistent with our definition. If multiple arms met the definition equally, we conservatively chose the one with the smallest PHQ‑9 improvement.

Blinded RCTs with participant blinding, investigator or assessor blinding, or double blinding were eligible for inclusion. Open‑label trials were excluded from this study. The impact of different blinding conditions was assessed by subgroup analysis and univariable regression. The blinded RCTs that used digital placebos as comparators were identified using the following approach. A total of 8 databases, including MEDLINE, Scopus, Web of Science, PsycINFO, CINAHL, Cochrane Central Register of Controlled Trials, ClinicalTrials.gov, and ISRCTN, were systematically searched with the relevant keywords: (random* OR RCT) AND (blind OR blinded OR placebo OR sham) AND (digital OR mhealth OR ehealth OR app OR apps OR application* OR smartphone OR mobile OR online OR computer-based OR web-based OR internet-based OR internet-delivered OR “virtual reality” OR VR OR “augmented reality” OR AR OR wearable* OR game* OR gamifi* OR “artificial intelligence” OR “machine Learning” OR “AI” OR “deep learning”) AND (PHQ9 OR PHQ-9 OR “Patient health questionnaire-9”) in November 2025. The search language was limited to English.

After excluding the duplicates and nonjournal records (preprints, dissertations, trial registry reports, and conference proceedings) on the reference management software, the papers without results (eg, protocols and reviews) and those evaluating drugs and supplements were excluded by title and abstract screening. Trials identified through clinical trial registration systems were included in the full‑text review when the results were available. In the full-text review, the papers that did not evaluate software (eg, transcranial direct current stimulation and electronic acupuncture), those without digital placebos as comparators (eg, waitlist and treatment-as-usual), were excluded. The papers that did not include PHQ-9 as depressive symptom scores, duplicate trials, and the trials with inappropriate study designs (eg, open-label trial, nonrandomized trial, and trial for children or adolescents younger than 18 years) were also excluded. Trial selection was performed independently by TH and RT, and the discrepancies were resolved through consensus meetings.

### Data Analysis

The following information was extracted from the selected papers for this systematic review and meta-analysis: (1) general information (author, clinical trial registration number, and publication year), (2) demographic characteristics (target population and mean age), (3) placebo device characteristics (placebo delivery type and placebo approach), (4) study design information (total number of patients, blindness, treatment period, number of groups, and study region), and (5) outcome information (end point type, mean, and SD data of PHQ-9 at pre- and postintervention for control arms).

The target population was classified into 3 groups in this study. The group of “Primary psychiatric disorder” included the participants who had been diagnosed with psychiatric disorders based on clinical guidelines such as *DSM-5* (*Diagnostic* *and Statistical Manual of Mental Disorders, Fifth Edition*) at screening [20]. The group of “Other diseases” included the patients with nonpsychiatric disorders such as cancer or pain. The group of other participants was classified as “Nonpatients.”

The delivery type was classified into the following 4 categories by delivery modality based on previous research as “Web/Mobile,” “Web,” “Mobile,” “Computer,” “Virtual Reality/Augmented Reality,” and “Wearable.” “Web/Mobile” refers to products available in both web and mobile versions [14,19]. The digital placebo approach was classified according to how active components are modified and was categorized into 4 types based on previous research [11,14,19]. “Replaced” is an approach that replaces the active component with an inactive or neutral component, for example, replacing the component of dialectical behavior therapy with nontherapeutic information of health and lifestyle topics. “Removed” is an approach that simply removes active component, for example, a locked version only with baseline survey and a short introduction video. “Unrelated” is an approach that the active component is replaced by a different active component which is unrelated, for example, trauma-focused procedures in active intervention were replaced with stress-reduction training. “Less” is an approach that is less-intense version of the active component, for example, deleting selected components of cognitive behavioral therapy for insomnia while providing sleep hygiene education.

Blindness was classified into 3 types. “Double” indicates a double-blinded trial. “Single-I” refers to a single-blinded trial that investigators or assessors were blinded but participants were not. “Single-P” refers to a single-blinded trial that participants were blinded but investigators or assessors were not. The postintervention data at the end of treatment or the closest available were adopted in each trial although the long-term effects after the completion of the treatment were evaluated in some selected trials. If the means or the SDs of PHQ-9 were not reported in the papers, the corresponding author was contacted by email. If no reply was obtained, the data were imputed by converting available SEs or 95% CIs into SDs as recommended by the Cochrane group [21]. The trials without sufficient information for meta-analysis despite these efforts were excluded from this study. The data extraction from the selected papers was conducted by TH and RT independently, and the discrepancies were resolved through consensus meetings. We adopted Hedges *g* to estimate the magnitude of improvement in digital placebo arms because the trials with small sample size were included in this study. A random-effects model was used by assuming high heterogeneity. In this study, Hedges *g* represented within-group standardized mean PHQ-9 improvement in digital placebo arms, and the value indicates greater symptom improvement in digital placebo arms. Hedges *g* was calculated directly from the reported pre- and postintervention means and their SDs. Positive τ² was estimated using maximum likelihood. The publication bias was evaluated by Begg modified funnel plot, the Duval and Tweedie trim-and-fill procedure, and the Egger regression intercept. Risk-of-bias assessment was performed by using the revised Cochrane risk-of-bias tool for randomized trials (RoB2) [21]. Sensitivity analysis was conducted by excluding the trials with high risk of bias and by excluding the papers that required imputation for the means or the SDs. A sensitivity analysis limited to the trials where PHQ-9 was a primary end point was also conducted. Leave-one-out sensitivity analysis was also conducted to examine the robustness of the findings. Subgroup analyses for categorical variables and meta-regressions for categorical and continuous variables were conducted to explore the reasons for heterogeneity and the potential moderators for improvement in digital placebo arms. Variables were considered statistically significant when the *P* values were <.05 in both the subgroup analyses and the meta-regressions. This criterion was used as a conservative approach to enhance the robustness of the moderator assessment by ensuring consistency across methods with different assumption and statistical power. Variables that were statistically significant in the univariable regression were entered into the multivariable regression. All statistical analyses were performed using meta package with R (version 4.4.2; R Foundation for Statistical Computing).

## Results

### Trial Selection for Systematic Review

A total of 1083 records were identified as potentially relevant. Following title and abstract screening, 215 papers were identified. After full-text review, 29 papers and 30 trials with 5680 participants were included for the systematic review (Figure 1) [22-50]. The population in 1 paper was divided into high-risk group and low-risk group according to the mental risk the authors defined. As the 2 groups were mutually exclusive and no participants were included in both groups, we regarded them as 2 independent trials [23]. The trials in this systematic review and meta-analysis were listed in Table 1 [22-50]. The trials excluded after full-text review are listed in Multimedia Appendix 2.

**Figure 1:**
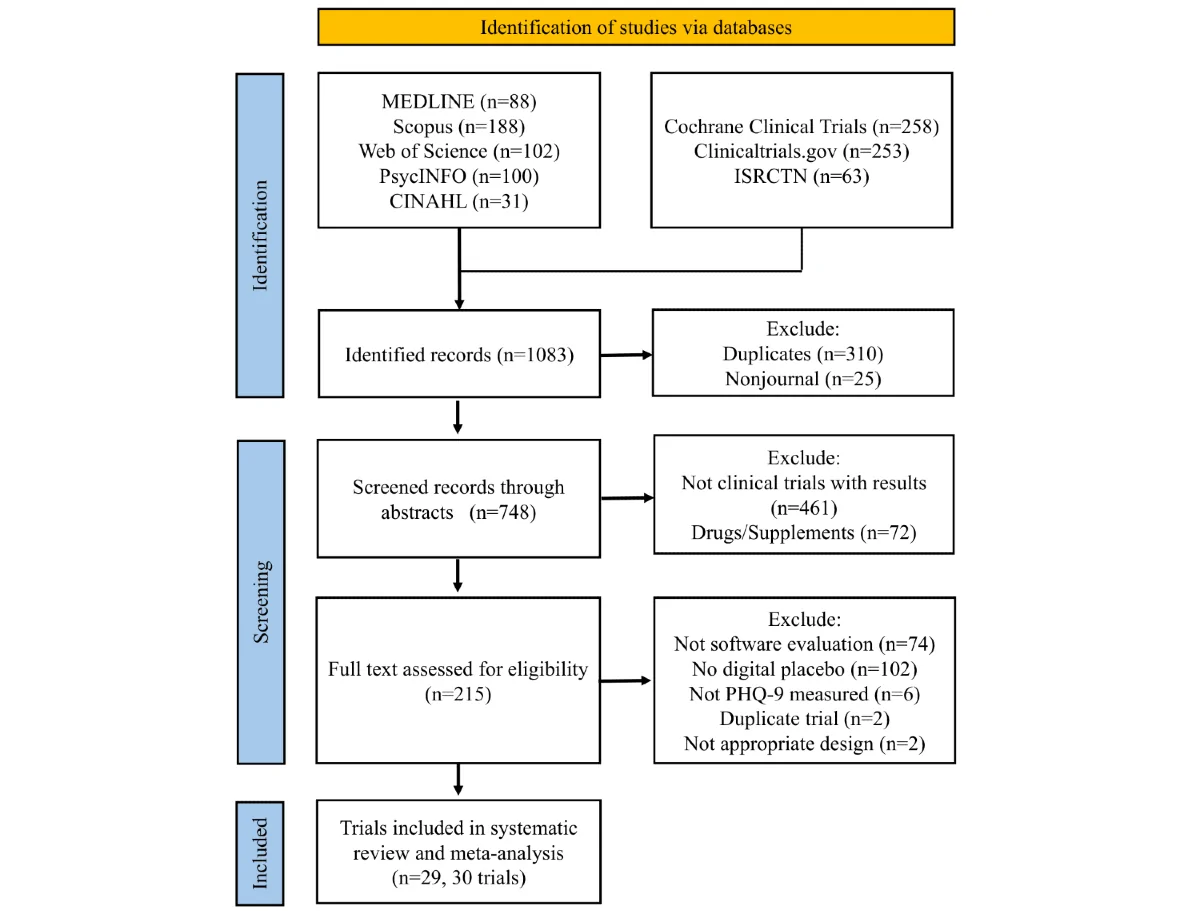
PRISMA (Preferred Reporting Items for Systematic Reviews and Meta-Analyses) chart. The PRISMA chart for systematic review and meta-analysis was shown with the number of records included or excluded at each step. PHQ-9: Patient Health Questionnaire-9.

**Table 1:** The trials included in the systematic review and meta-analysis.

Study	Delivery type	Approach	Blindness	Target population	End point	Mean age (years)	Treatment (day)	Group, n	Total, N	Region	Risk
Glozier et al [22], 2013	Web	Replaced	Double^a^	Other diseases	Primary^b^	58.4	84	2	562	APAC^c^	Low
Musiat et al [23]: high (2014)	Web	Unrelated	Single-P^d^	Nonpatients	Primary	21.0^a^	84	2	181	EMEA^e^	High
Musiat et al [23]: low, 2014	Web	Unrelated	Single-P	Nonpatients	Primary	21.0^a^	84	2	859	EMEA^e^	High
Christensen et al [24], 2016	Web	Replaced	Single-I^f^	Psychiatric disorder	Primary	42.5	42	2	1149	APAC^c^	Some concerns
Clarke et al [25], 2019	Web/Mobile	Removed	Double	Other diseases	Secondary^g^	57.7	56	2	780	APAC^c^	Low
Wittekind et al [26], 2019	Computer	Removed	Single-P	Nonpatients	Secondary	33.7	56	2	141	EMEA^e^	Some concerns
Delbaere et al [27], 2021	Mobile	Unrelated	Single-I	Non-patients	Secondary	77.7	730	2	503	APAC^c^	Some concerns
Dingwall et al [28], 2021	Mobile	Replaced	Single-P	Other diseases	Primary	53.9	21	3	156	APAC^c^	Low
Heim et al [29], 2021	Mobile	Removed	Single-P	Nonpatients	Primary	26.4	35	2	138	EMEA^e^	Some concerns
Hirsch et al [30], 2021	Web	Replaced	Single-P	Psychiatric disorder	Secondary	35.7	30	2	230	EMEA^e^	Some concerns
Cuijpers et al [31], 2022	Web/Mobile	Replaced	Single-P	Nonpatients	Primary	27.1	56	2	680	EMEA^e^	Some concerns
De Kock et al [32], 2021	Mobile	Replaced	Single-P	Nonpatients	Primary	NS^h^	28	3	169	EMEA^e^	Low
He et al [33], 2022	Web	Unrelated	Double	Nonpatients	Primary	18.6	7	3	148	APAC^c^	Low
Smidt et al [34], 2022	Mobile	Removed	Single-P	Nonpatients	Secondary	19.3	14	2	55	NA^i^	Low
Torok et al [35], 2022	Mobile	Replaced	Double	Nonpatients	Secondary	21.7	42	2	455	APAC^c^	Low
Alon et al [36], 2023	Web	Removed	Double	Psychiatric disorder	Secondary	38.9	28	2	60	EMEA^e^	Low
Davenport and Werner [37], 2023	Wearable	Removed	Double	Psychiatric disorder	Secondary	45.6	30	2	72	NA^i^	Some concerns
Ehlers et al [38], 2023	Web	Unrelated	Single-I	Psychiatric disorder	Secondary	35.8	84	3	217	EMEA^e^	Some concerns
Graessel et al [39], 2024	Computer	Replaced	Double	Nonpatients	Secondary	73.5	180	2	89	EMEA^e^	Low
Mak et al [40], 2024	Web/Mobile	Replaced	Single-I	Nonpatients	Primary	28.2	42	3	256	APAC^c^	Some concerns
Nakamura et al [41], 2024	Mobile	Replaced	Single-I	Nonpatients	Primary	65.3	42	2	454	LATAM^j^	Some concerns
Scazufca et al [42], 2024	Mobile	Replaced	Single-I	Nonpatients	Primary	65.1	42	2	603	LATAM^j^	Some concerns
Shin et al [43], 2024	Mobile	Less	Single-P	Psychiatric disorder	Secondary	40.5	42	2	98	APAC^c^	Some concerns
Vereschagin et al [44], 2024	Mobile	Removed	Single-I	Nonpatients	Primary	20.0^k^	30	2	1489	NA^i^	Some concerns
Cartwright et al [45], 2025	Web	Replaced	Single-P	Other diseases	Primary	48.0^k^	28	2	67	EMEA^e^	High
Chen et al [46], 2025	Mobile	Replaced	Single-I	Psychiatric disorder	Secondary	22.2	42	2	708	APAC^c^	Some
Lee et al [47], 2025	Mobile	Replaced	Double	Psychiatric disorder	Secondary	36.0	42	2	120	APAC^c^	High
Rothman et al [48], 2025	Mobile	Replaced	Double	Psychiatric disorder	Secondary	42.1	42	2	386	NA^i^	Low
Torok et al [49], 2025	Mobile	Replaced	Double	Nonpatients	Secondary	19.7	120	3	692	APAC^c^	Low
Tsubono and Mitoku [50], 2025	Web/Mobile	Replaced	Single-P	Nonpatients	Primary	NS^h^	28	2	375	APAC^c^	Some concerns

^a^Double: both participants and investigators are blinded.

^b^Primary: PHQ-9 was primary end point.

^c^APAC: Asia-Pacific.

^d^Single-P: only participants are blinded.

^e^EMEA: Europe, Middle East, and Africa.

^f^Single-I: only investigators or assessors are blinded.

^g^Secondary: PHQ-9 was secondary end point.

^h^NS: Not specified.

^i^NA: North America.

^j^LATAM: Latin America.

^k^Median.

### Characteristics of the Included Trials for Systematic Review and Meta-Analysis

The characteristics of the identified trials are summarized in Multimedia Appendix 3. All the selected trials were parallel-group RCTs in a single country. Overall, 80% of the trials were published in 2021 or later. More than half of the trials were targeted “nonpatients” group. The “Primary psychiatric disorder” group included patients with major depressive disorder, anxiety, insomnia, and posttraumatic stress disorder. The weighted mean age was 38.2 years among the 28 trials that reported it. Mobile platform was mostly used as delivery type and 63.3% of the identified trials adopted “Mobile” or “Web/Mobile.” The placebo approach of “Replaced” represented 56.7% of the included trials. Overall, 80% of the identified trials were 2-arm RCTs. Blindness was almost evenly divided into 3 groups. The weighted mean treatment period of the identified trials was 82.1 days ranging from 7 days to 730 days. The mean sample size of the identified trials was 396.5 ranging from 55 to 1489. Many trials were conducted in Asia-Pacific or Europe, Middle East, and Africa regions. None of the selected trials were multiregional clinical trials (MRCTs). Four trials were categorized as “High risk” with the risk-of-bias assessment.

### Magnitude of Improvement in Digital Placebo Arms

The pooled effect size for all the included trials was Hedges *g*=0.44 (95% CI 0.29-0.59) with high heterogeneity (*I*^2^=93.2%, τ²=0.14) as shown in Figure 2 [22-50]. Positive Hedges *g* value shows greater PHQ-9 improvement within the digital placebo arms. The Egger test did not indicate publication bias (*P*=.55). The funnel plot did not show any substantial publication bias visually, and the Duval and Tweedie trim-and-fill method did not indicate any unpublished trials (Multimedia Appendix 4). The summary results of risk of bias and the detailed results in each trial are shown in Figure 3. “Some concerns” judgments were driven by “Deviations from the intended interventions” and “Measurement of the outcome” while “Selection of reported result” accounted for the most “High risk” judgments. The results of risk-of-bias assessment for each trial are shown in Multimedia Appendix 5 [22-50]. The improvement in the placebo arms remained statistically significant after excluding 4 trials with “High risk” (Hedges *g*=0.48, 95% CI 0.32-0.64, *I*^2^=93.7%, τ²=0.15), after excluding 8 trials that required the imputation for the SDs (Hedges *g*=0.48, 95% CI 0.35-0.61, *I*^2^=86.5%, τ²=0.07), and after excluding 15 trials where PHQ-9 was measured as a secondary end point (Hedges *g*=0.36, 95% CI 0.20-0.52, *I*^2^=87.4%, τ²=0.07). Leave-one-out sensitivity analyses indicated that the exclusion of any single trial did not change the statistical significance of the pooled effect.

**Figure 2:**
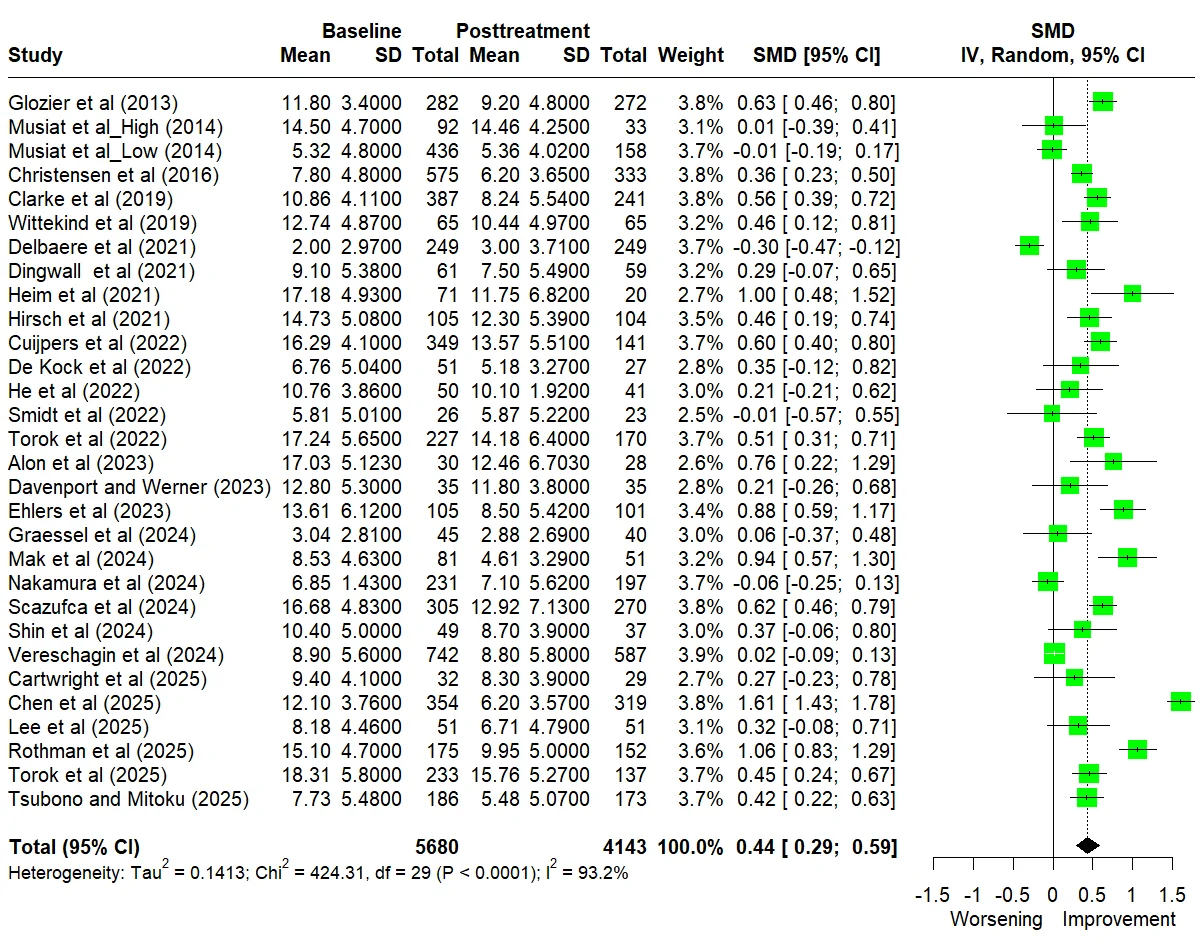
Forest plot of included trials in this meta-analysis [22-50]. IV: inverse variance method; Random: random effects model. SMD: standardized mean difference.

**Figure 3:**
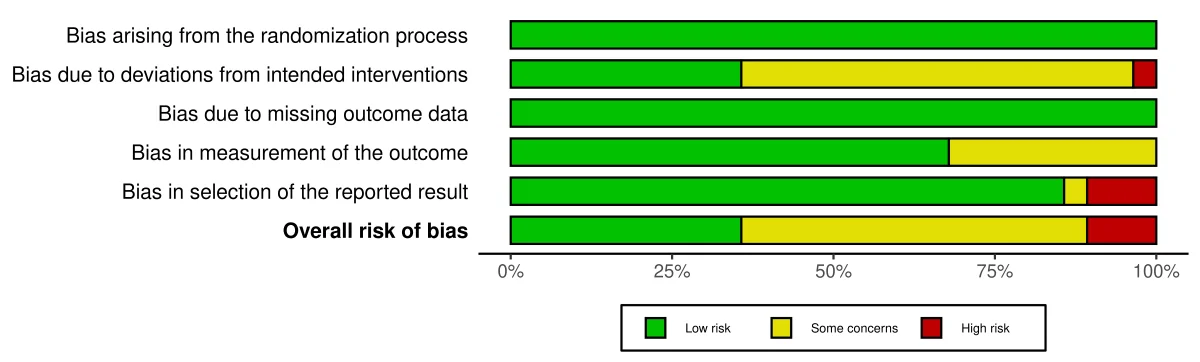
Summary results of risk-of-bias assessment of included trials using ROB2 (revised version of the Cochrane Risk-of-Bias tool for randomized trials).

### Results of Subgroup Analyses and Meta-Regressions

Subgroup analyses on categorical variables showed the statistically significant subgroup differences for target population (*P*=.02) and risk of bias (*P*<.001) (Table 2). The “Primary psychiatric disorder” group in target population showed larger effect size than the other 2 groups, indicating a moderate-to-large placebo effect (Hedges *g*=0.69, 95% CI 0.40-0.99). The effect size of the trials categorized as “High risk” by risk-of-bias assessment was much smaller than that of other trials. Among the 4 trials rated as “High risk,” any consistent patterns in population, baseline PHQ-9, digital placebo features, or end point status were identified.

**Table 2:** Results of subgroup analyses.

Group and subgroup		Value, n	Hedges *g* (95% CI)	*I*^2^ value (%)	*P* value	
**Target population**						.02
	Nonpatients	17	0.30 (0.13 to 0.46)	88.6		
	Other diseases	4	0.55 (0.44 to 0.66)	23.2		
	Primary psychiatric disorder	9	0.69 (0.40 to 0.99)	94.6		
**Placebo delivery type**						.84
	Computer	1	0.47 (0.12 to 0.81)	N/A^a^		
	Mobile	13	0.48 (0.20 to 0.75)	96.7		
	Wearable	1	0.21 (–0.26 to 0.68)	N/A^a^		
	Web	9	0.39 (0.20 to 0.59)	81.7		
	Web/Mobile	6	0.49 (0.31 to 0.67)	65.3		
**Placebo approach**						.29
	Less	1	0.37 (–0.06 to 0.80)	N/A^a^		
	Replaced	17	0.54 (0.35 to 0.72)	92.9		
	Removed	7	0.40 (0.16 to 0.65)	86.6		
	Unrelated	5	0.15 (–0.21 to 0.51)	91.6		
**Blindness**						.26
	Double	9	0.54 (0.37 to 0.71)	72.4		
	Single-I	9	0.47 (0.10 to 0.84)	97.6		
	Single-P	12	0.35 (0.20 to 0.50)	66.6		
**End point type**						.31
	Primary	15	0.36 (0.20 to 0.52)	87.4		
	Secondary	15	0.51 (0.27 to 0.75)	94.8		
**Study** **region**					.85	
	APAC^b^	13	0.50 (0.26 to 0.73)	95.2		
	EMEA^c^	11	0.43 (0.23 to 0.62)	79		
	LATAM^d^	2	0.28 (–0.19 to 0.80)	96.5		
	NA^e^	4	0.34 (–0.11 to 0.80)	95.3		
**Risk of bias**						<.001
	Low	11	0.49 (0.32 to 0.65)	71.6		
	Some concerns	15	0.50 (0.26 to 0.74)	96		
	High	4	0.06 (–0.08 to 0.21)	0		

^a^N/A: Not applicable.

^b^APAC: Asia-Pacific.

^c^EMEA: Europe, Middle East, and Africa.

^d^LATAM: Latin America.

^e^NA: North America.

Univariable regression on categorical and continuous variables indicated that “Primary psychiatric disorder” group in target population (*P*=.008), treatment period (*P*=.03), and baseline PHQ-9 score (*P*<.001) were the moderators of the improvement in digital placebo arms (Table 3). Because treatment duration ranged widely across trials (7-730 days), we additionally performed a sensitivity analysis excluding the trial with a 730-day duration. The univariable regression showed that association between treatment period and improvement in placebo arms was no longer statistically significant (*P*=.63).

Multivariable regression suggested that “Primary psychiatric disorder“ group in target population (*β*=.34, 95% CI 0.09-0.59; *P*=.008) and baseline PHQ-9 score (*β*=.05, 95% CI 0.02-0.07; *P*<.001) were major and independent contributing factors of the high heterogeneity (*I*^2^=82.1%, τ²=0.07, *R*^2^=51.5%) while treatment period was not an independent moderator of improvement in digital placebo arms (*β*=–.004, 95% CI –0.001 to 0.0005; *P*=.43).

**Table 3:** Results of univariable regression.

Variable and level			Value, n		Coefficient (95% CI)		*I*^2^ value (%)		*P* value
**Publication year**									
	N/A^a^	30		0.02 (–0.01 to 0.07)		90.3		.19	
**Target population**									
	Nonpatients	30		Reference		88.2		N/A	
	Other diseases	N/A		0.16 (–0.24 to 0.56)		N/A		.43	
	Primary psychiatric disorder	N/A		0.40 (0.10 to 0.70)		N/A		.008	
**Age (years)**									
	N/A	28		–0.006 (–0.01 to 0.00)		90.9		.18	
**Placebo delivery type**									
	Computer	30		Reference		91			
	Mobile	N/A		0.09 (–0.83 to 0.85)		N/A		.98	
	Wearable	N/A		–0.25 (–1.44 to 0.94)		N/A		.68	
	Web	N/A		–0.07 (–0.92 to 0.78)		N/A		.87	
	Web/Mobile	N/A		–0.01 (–0.88 to 0.86)		N/A		.98	
**Placebo approach**									
	Less	30		Reference		89.2		N/A	
	Removed	N/A		0.04 (–0.82 to 0.90)		N/A		.92	
	Replaced	N/A		0.17 (–0.66 to 0.99)		N/A		.69	
	Unrelated	N/A		–0.22 (–1.09 to 0.65)		N/A		.62	
**Total** **number of patients**									
	N/A	30		–0.0000 (–0.0005 to 0.0004)		90.5		.84	
**Blindness**									
	Double	30		Reference		90.5		N/A	
	Single-I	N/A		–0.05 (–0.42 to 0.31)		N/A		.78	
	Single-P	N/A		–0.17 (–0.52 to 0.19)		N/A		.35	
**Treatment period**									
	N/A	30		–0.001 (–0.002 to –0.0001)		89.3		.03	
**Number of groups**									
	N/A	30		0.11 (–0.26 to 0.48)		91		.56	
**Study region**									
	APAC^b^	30		Reference		N/A			
	EMEA^c^	N/A		–0.06 (–0.39 to 0.26)		N/A		.70	
	LATAM^d^	N/A		–0.21 (–0.78 to 0.36)		N/A		.47	
	NA^e^	N/A		–0.15 (–0.60 to 0.31)		N/A		.53	
**End point type**									
	Primary	30		Reference		90.5		N/A	
	Secondary	N/A		0.15 (–0.14 to 0.43)		N/A		.31	
**Baseline PHQ-9^f^ score**									
	N/A	30		0.05 (0.02 to 0.08)		87.1		<.001	
**Risk of bias**									
	High	30		Reference		90.1		N/A	
	Low	N/A		0.33 (–0.13 to 0.79)		N/A		.16	
	Some concerns	N/A		0.36 (–0.08 to 0.80)		N/A		.11	

^a^N/A: not applicable.

^b^APAC: Asia-Pacific.

^c^EMEA: Europe, Middle East, and Africa.

^d^LATAM: Latin America.

^e^NA: North America.

^f^PHQ-9: Patient Health Questionnaire-9.

## Discussion

### Principal Results

This study revealed that digital placebos have actively started to be used as comparators in recent DTx clinical trials. The most common design was 2-arm, parallel-group RCTs conducted in a single country, adopting “Replaced” and “Mobile” as the placebo approach and delivery type, respectively. A small-to-moderate statistically significant improvement was observed in digital placebo arms with high heterogeneity in the pooled analysis of all the included trials. No substantial publication bias was indicated and the findings were generally robust in sensitivity analyses. Both subgroup analyses and meta-regressions indicated that “Primary psychiatric disorder” group in target population and baseline PHQ-9 score were major independent moderators of improvement in digital placebo arms which accounted for a substantial portion of the heterogeneity.

### Comparison With Prior Work

The characteristics of the RCTs included in this study were similar to those of our previous study on GAD symptom scores, whereas “Web” delivery type had been the most common [14]. The proportion and variety of digital placebos used in clinical trials may evolve with the advancement of digital technology in the future [51]. Unlike the clinical trials of pharmacotherapy, no MRCTs were conducted in the selected trials. Lack of internationally harmonized regulatory framework, immature local reimbursement systems, small company size and limited resources, predominance of pilot studies, cultural adaptation and platform localization, and language barriers in translation might be the hurdles for conducting MRCTs [52-54].

The “Primary psychiatric disorder” group in target population in this study showed larger placebo effect (Hedges *g*=0.69) that exceeded our previous finding on GAD scores in the same population (Hedges *g*=0.52) [14]. Those results were consistent with the previous research in pharmacotherapy demonstrating that major depressive disorder showed the largest placebo effect across 9 psychiatric disorders [55]. The finding that higher baseline PHQ‑9 score exhibited larger improvement in digital placebo arms aligned with patient-level evidence from antidepressant trials showing that baseline depressive severity was associated with larger placebo effects as well as larger treatment effects [56].

The findings that “Primary psychiatric disorder” group in target population and baseline PHQ-9 score were major independent moderators for improvement in digital placebo arms were in line with our previous study [14]. This study provided a more methodologically rigorous estimate of improvement in digital placebo arms by restricting symptom score to PHQ-9. These results suggest that improvement in digital placebo arms may extend across psychiatric symptoms, but careful consideration is needed. For example, “Primary psychiatric disorder” group in this study was heterogeneous and may differ clinical trajectory, expectancy, treatment context, and likelihood of spontaneous improvement. The moderation effect of target population may also reflect multiple clinical and contextual factors rather than a single mechanism. In addition, the moderating role of baseline PHQ‑9 score should be interpreted with caution, as higher baseline scores allow greater room for improvement and may partly reflect mathematical coupling or regression to the mean rather than expectancy-driven mechanism alone [56,57].

Some of the other variables that appeared statistically significant in subgroup analyses or meta-regressions were not definitive. Risk of bias was statistically significant in subgroup analyses but did not remain significant in meta-regressions. The smaller effect size observed in the “high risk” group was largely attributable to 1 trial in which PHQ-9 score in the placebo arms was worsened posttrial. Because “Computer” and “Wearable” in placebo delivery type and “Less” in placebo approach included only 1 trial, the corresponding subgroup estimates are descriptive only and no conclusions can be drawn for these categories. Although the univariable regression indicated that treatment period was a moderator, this association disappeared after excluding the trial, the treatment duration of which was 730 days, indicating that the apparent effect was driven by this extreme outlier. Consistent with this, multivariate regression indicated that treatment period was not an independent moderator. Further research is needed to determine whether these variables function as moderators. Although digital placebo effects are also considered to be influenced by sociocultural and technological factors more than pharmacotherapy, the variables assessed in this study such as age, placebo delivery type, and study region were not identified as moderators [58]. These variables may capture only limited aspects of sociocultural or technological contexts, and robust clinical trial designs such as blinded RCTs may attenuate their influence, but further research with more granular contextual measures is needed to clarify how they interact with digital placebo effects.

It is also important to recognize mechanisms that underline digital placebo responses. Expectancy effects, well-known in pharmacotherapy for depressive disorders, may be observable in digital environment, where the functions such as structured user interfaces, self-monitoring, push notifications, personalization, and interactive elements may create a convincing therapeutic experience even in the absence of active ingredients [13]. Furthermore, neurochemical activation and neurological changes observed in pharmacological placebo responses may also be triggered by digital placebos through the expectancy and engagement processes [59,60]. However, these interpretations remain speculative and are not supported by the findings of this study. They should be considered hypothesis-generating, and further research is needed to clarify the underlying scientific validity and the mechanisms of digital placebo effects.

The findings of this study have several implications for the designs of future clinical trials with digital placebos. Our results indicate that PHQ-9 improvement in digital placebo arms is not negligible and should be incorporated into trial design. The moderators identified in this study should also be considered when estimating expected improvement in control arms. Concurrently, the pooled improvement in digital placebo arms identified in this study should not be used as a universal assumption because of the substantial heterogeneity. Population-specific and context-specific estimates are more appropriate not only for sample size planning but also for the broader designs of powered DTx clinical trials, alongside other conceptual and methodological factors that may influence digital placebo effects.

Conducting a pilot trial with target population may address some practical uncertainties discussed previously before the confirmatory trial [61]. The pilot trial will allow investigators to identify which digital placebo conditions can feasibly support blinding, to assess the credibility, and to refine the intensity accordingly. The study also provides empirical information on expectancy, adherence, and other conceptual and methodological factors. These data may provide insights into the necessity of additional features such as notifications and human support, and sample size requirements for the confirmatory trial. Complementing these practical study design considerations, structured reporting of comparator content, appearance, interactivity, notifications, human support, engagement metrics, and participant credibility ratings would advance digital placebo research.

### Limitations

There are several limitations in this study. This study included only adults aged 18 years or older. Further studies are needed to investigate digital placebo effects for children and adolescents. Participant-level data were not available, and patient-level moderators such as sex, age, severity distributions, comorbidities, digital literacy, expectancy, credibility, engagement, adherence, and concomitant treatment were not evaluated.

We defined digital placebos, placebo delivery type, and placebo approach based on previous research, given that no universal definition exists. Under the definition, part of active interventions such as low-intensity intervention by another classification might be included in this study. Conceptual relationship between delivery type and placebo approach was not investigated either.

We included trials in which PHQ-9 was measured as a secondary end point and any mode of PHQ-9 administration was accepted. We cannot exclude the possibility that those factors influenced PHQ-9 responses. Because our search strategy required PHQ-9 terms, relevant trials may not have been retrieved if PHQ-9 was not mentioned in searchable fields. We did not consider the long-term effects of digital placebos. We adopted the postintervention data at the end of treatment or the closest available timepoint to standardize the conditions across trials.

Several trials were single-blinded, with investigators or assessors blinded but not participants. The variability may contribute to the improvement in placebo arms. Improvement observed in placebo arms cannot be separated from the natural course of depressive symptoms because no-treatment controls were not available. Publication bias assessment by using Begg modified funnel plots, Egger test, and Duval and Tweedie trim‑and‑fill procedures has limited sensitivity under high heterogeneity. The review was restricted to publications in English and may exclude the relevant trials from non–English-speaking regions.

### Conclusions

A small-to-moderate and statistically significant PHQ-9 improvement in digital placebo arms was identified in the pooled analysis of all the included trials with high heterogeneity. Subgroup analyses and meta-regressions indicated that both “Primary psychiatric disorder” in target population and baseline PHQ-9 score were major independent moderators of PHQ-9 improvement which accounted for a substantial proportion of heterogeneity. Our findings indicate that improvement in digital placebo arms is not negligible and should be incorporated into trial design. The moderators identified in this study will be useful when estimating expected improvement in control arms and planning adequately powered future DTx clinical trials, alongside other relevant conceptual and methodological factors that may influence digital placebo effects.

## Appendix

Multimedia Appendix 1 PRISMA (Preferred Reporting Items for Systematic Reviews and Meta-Analyses) checklist.

Multimedia Appendix 2 Trials excluded from this study after full-text review.

Multimedia Appendix 3 Characteristics of the trials included in this study.

Multimedia Appendix 4 Funnel plot.

Multimedia Appendix 5 Results of risk-of-bias assessment in each study.

## Abbreviations

DSM-5: Diagnostic and Statistical Manual of Mental Disorders, Fifth Edition
DTx: digital therapeutics
GAD: generalized anxiety disorder
MRCT: multiregional clinical trial
PHQ-9: Patient Health Questionnaire-9
PICO: Population, Intervention, Comparison, Outcome
PRISMA: Preferred Reporting Items for Systematic Reviews and Meta-Analyses
PROSPERO: International Prospective Register of Systematic Reviews
RCT: randomized controlled trial
