# Supplementary material for: Magnitude and Moderators of Depressive Symptom Improvement in Digital Placebo Arms: Systematic Review and Meta-Analysis
Source: J Med Internet Res. 2026 Oct 9;28:e100426. doi: 10.2196/100426 (PMC13695001; doi:10.2196/100426)
Supplement: Supplementary file 2 [file jmir_v28i1e100426_app2.docx]

Multimedia Appendix 2. Trials Excluded from This Study after Full-text Review

| Author | Reason | Author | Reason |
| --- | --- | --- | --- |
| Kroenke et al, 2010 [1] | No digital placebo | Islam et al, 2019 [33] | No digital placebo |
| Dwight-Johnson et al, 2011 [2] | No digital placebo | Kehle-Forbes et al, 2019 [34] | No digital placebo |
| Campbell-Sills et al, 2012 [3] | No digital placebo | Miloff et al, 2019 [35] | No digital placebo |
| Moreno et al, 2012 [4] | No digital placebo | Niederkrotenthaler et al, 2019 [36] | Not PHQ-9 measured |
| Bombardier et al, 2013 [5] | No digital placebo | Peoples et al, 2019 [37] | No digital placebo |
| Houlihan et al, 2013 [6] | No digital placebo | van der Zweerde et al, 2019 [38] | No digital placebo |
| Chavira et al, 2014 [7] | No digital placebo | Zalta et al, 2019 [39] | Not software evaluation |
| Christensen, 2014 [8] | Not PHQ-9 measured | Armstrong et al, 2020 [40] | No digital placebo |
| Pickett et al, 2014 [9] | Not software evaluation | Ifejika et al, 2020 [41] | No digital placebo |
| Gilbody et al, 2015 [10] | No digital placebo | Koranyi et al, 2020 [42] | Not software evaluation |
| Schoene et al, 2015 [11] | No digital placebo | Kyle et al, 2020 [43] | No digital placebo |
| Hudson et al, 2016 [12] | No digital placebo | Philip et al, 2020 [44] | Not software evaluation |
| Moritz et al, 2016 [13] | No digital placebo | Rae Olmsted et al, 2020 [45] | Not software evaluation |
| Wald et al, 2016 [14] | Not appropriate design | Reeves et al, 2020 [46] | No digital placebo |
| Freeman et al, 2017 [15] | No digital placebo | Russell et al, 2020 [47] | No digital placebo |
| Hudson et al, 2017 [16] | No digital placebo | Sander et al, 2020 [48] | No digital placebo |
| Klein et al, 2017 [17] | No digital placebo | Segal et al, 2020 [49] | No digital placebo |
| Klein et al, 2017 [18] | No digital placebo | Baumeister et al, 2021 [50] | No digital placebo |
| Rollman et al, 2017 [19] | Not software evaluation | Elbogen et al, 2021 [51] | Not software evaluation |
| Thomas et al, 2017 [20] | Not software evaluation | Fletcher et al, 2021 [52] | No digital placebo |
| Ferrari et al, 2018 [21] | Not software evaluation | Klein et al, 2021 [53] | Not software evaluation |
| Gavriloff et al, 2018 [22] | Not PHQ-9 measured | Mclean et al, 2021 [54] | No digital placebo |
| Kennard et al, 2018 [23] | No digital placebo | Meyerhoff et al, 2021 [55] | No digital placebo |
| Menant et al, 2018 [24] | No digital placebo | Morriss et al, 2021 [56] | No digital placebo |
| O’Moore et al, 2018 [25] | No digital placebo | Nolan et al, 2021 [57] | Not PHQ-9 measured |
| Rollman et al, 2018 [26] | No digital placebo | Nolte et al, 2021 [58] | No digital placebo |
| Scheider et al, 2018 [27] | No digital placebo | Owens et al, 2021 [59] | Not software evaluation |
| Zagorscak et al, 2018 [28] | No digital placebo | Raevuori et al, 2021 [60] | No digital placebo |
| Cunningham et al, 2019 [29] | No digital placebo | Scott et al, 2021 [61] | Not software evaluation |
| Donker et al, 2019 [30] | No digital placebo | Seal et al, 2021 [62] | Not software evaluation |
| Forand et al, 2019 [31] | No digital placebo | Yang et al, 2021 [63] | No digital placebo |
| Grafe et al, 2019 [32] | No digital placebo | Boege et al, 2022 [64] | No digital placebo |

| Author | Reason | Author | Reason |
| --- | --- | --- | --- |
| Cuijpers et al, 2022 [65] | Duplicated study | Kleinau et al, 2024 [97] | Not software evaluation |
| Kessler et al, 2022 [66] | No digital placebo | Lee et al, 2024 [98] | Not software evaluation |
| Langergaard et al, 2022 [67] | No digital placebo | Li et al, 2024 [99] | Not software evaluation |
| Mathiasen et al, 2022 [68] | No digital placebo | Liang et al, 2024 [100] | Not software evaluation |
| Matsumoto et al, 2022 [69] | No digital placebo | Maulik et al, 2024 [101] | No digital placebo |
| Oslin et al, 2022 [70] | Not software evaluation | O’Neil et al, 2024 [102] | Not software evaluation |
| Paul et al, 2022 [71] | No digital placebo | Philip et al, 2024 [103] | No digital placebo |
| Zheng et al, 2022 [72] | No digital placebo | Picariello et al, 2024 [104] | No digital placebo |
| Benjet et al, 2023 [73] | No digital placebo | Safiah et al, 2024 [105] | Not software evaluation |
| Connell et al, 2023 [74] | No digital placebo | Shi et al, 2024 [106] | No digital placebo |
| Grobelski et al, 2023 [75] | No digital placebo | Song et al, 2024 [107] | Not software evaluation |
| Hllrigel-Holderbaum et al, 2023 [76] | No digital placebo | Woolf et al, 2024 [108] | Not software evaluation |
| Husain et al, 2023 [77] | Not software evaluation | Xiang et al, 2024 [109] | No digital placebo |
| Jelinek et al, 2023 [78] | No digital placebo | Xu et al, 2024 [110] | Not software evaluation |
| Kerber et al, 2023 [79] | No digital placebo | Yatziv et al, 2024 [111] | No digital placebo |
| Lester et al, 2023 [80] | Not software evaluation | Yue et al, 2024 [112] | No digital placebo |
| Lu et al, 2023 [81] | No digital placebo | Akturk et al, 2025 [113] | Not software evaluation |
| McGillivary et al, 2023 [82] | Duplicated study | Assmann et al, 2025 [114] | Not software evaluation |
| Mide et al, 2023 [83] | No digital placebo | Cheng et al, 2025 [115] | Not software evaluation |
| Moritz et al, 2023 [84] | No digital placebo | Fleischhauer et al, 2025 [116] | No digital placebo |
| Morriss et al, 2023 [85] | Not software evaluation | Hentati et al, 2025 [117] | Not PHQ-9 measured |
| Poole et al, 2023 [86] | No digital placebo | Herrero et al, 2025 [118] | No digital placebo |
| Stwart et al, 2023 [87] | No digital placebo | Kern et al, 2025 [119] | No digital placebo |
| Strauss et al, 2023 [88] | Not software evaluation | Kusumadewi et al, 2025 [120] | No digital placebo |
| Werner-Seidler et al, 2023 [89] | Not PHQ-9 measured | Lee et al, 2025 [121] | No digital placebo |
| Bauer et al, 2024 [90] | No digital placebo | Leung et al, 2025 [122] | No digital placebo |
| Daros et al, 2024 [91] | No digital placebo | Morriss et al, 2025 [123] | Not software evaluation |
| Gata et al, 2024 [92] | No digital placebo | Nilkantham et al, 2025 [124] | No digital placebo |
| Gitaari et al, 2024 [93] | No digital placebo | O’Driscoll et al, 2025 [125] | No digital placebo |
| Guo et al, 2024 [94] | No digital placebo | Pieh et al, 2025 [126] | Not software evaluation |
| Jagayat et al, 2024 [95] | Not software evaluation | Poole et al, 2025 [127] | No digital placebo |
| Kim et al, 2024 [96] | No digital placebo | Prott et al, 2025 [128] | Not software evaluation |

| Author | Reason | Author | Reason |
| --- | --- | --- | --- |
| Ravitz et al, 2025 [129] | Not software evaluation | Tulbure et al, 2025 [136] | No digital placebo |
| Rotstein et al, 2025 [130] | Not software evaluation | Vall-Roque et al, 2025 [137] | Not software evaluation |
| Schmidt et al, 2025 [131] | No digital placebo | Yan et al, 2025 [138] | No digital placebo |
| Simpson et al, 2025 [132] | Not software evaluation | Yang et al, 2025 [139] | No digital placebo |
| Stadler et al, 2025 [133] | Not software evaluation | Zhang et al, 2025 [140] | No digital placebo |
| Taguchi et al, 2025 [134] | Not appropriate design | Saeed et al, 2026 [141] | Not software evaluation |
| Tong et al, 2025 [135] | No digital placebo |  |  |

| Trial registration number | Reason | Trial registration number | Reason |
| --- | --- | --- | --- |
| NCT04638673 | Not software evaluation | ISRCTN17755571 | Not software evaluation |
| NCT02853032 | Not software evaluation | ISRCTN18107122 | No digital placebo |
| NCT03562767 | Not software evaluation | ISRCTN57805470 | No digital placebo |
| NCT02685358 | No digital placebo | ISRCTN19674644 | Not software evaluation |
| NCT03922529 | Not software evaluation | ISRCTN52074801 | Not software evaluation |
| NCT05551195 | No digital placebo | ISRCTN13339678 | Not software evaluation |
| NCT04280562 | Not software evaluation | ISRCTN11853110 | Not software evaluation |
| NCT04527328 | Not software evaluation | ISRCTN18055968 | No digital placebo |
| NCT03431493 | Not software evaluation | ISRCTN18418155 | Not software evaluation |
| NCT04099511 | Not software evaluation | ISRCTN17917944 | Not software evaluation |
| NCT03267810 | Not software evaluation | ISRCTN11357117 | No digital placebo |
| NCT03509909 | Not software evaluation | ISRCTN10942585 | Not software evaluation |
| NCT04012853 | Not software evaluation | DRKS00008745 | No digital placebo |
| NCT04507867 | Not software evaluation | ChiCTR2000030670 | Not software evaluation |
| NCT03779048 | Not software evaluation | ChiCTR2300078321 | Not software evaluation |
| NCT03304587 | Not software evaluation | ChiCTR2300078682 | Not software evaluation |
| NCT04195685 | Not software evaluation | ChiCTR2400079852 | Not software evaluation |
| NCT04405089 | Not software evaluation | RBR-688c6mb | Not software evaluation |
| NCT04830033 | No digital placebo | RBR-2t7pt25 | Not software evaluation |
| ISRCTN14601588 | Not software evaluation | RBR-7466htj | Not software evaluation |
| ISRCTN14644939 | No digital placebo | RBR-4pgd3ct | Not software evaluation |
| ISRCTN91146045 | Not software evaluation | KCT0009382 | No digital placebo |
| ISRCTN64408867 | Not software evaluation |  |  |

**References**

1. Kroenke K, Theobald D, Wu J, Norton K, Morrison G, Carpenter J, et al. Effect of telecare management on pain and depression in patients with cancer: a randomized trial. JAMA. 2010;304(2):163-71. doi:10.1001/jama.2010.944.
2. Dwight-Johnson M, Aisenberg E, Golinelli D, Hong S, O'Brien M, Ludman E. Telephone-based cognitive-behavioral therapy for Latino patients living in rural areas: a randomized pilot study. Psychiatr Serv. 2011;62(8):936-42. doi:10.1176/ps.62.8.pss6208_0936
3. Campbell-Sills L, Sherbourne CD, Roy-Byrne P, Craske MG, Sullivan G, Bystritsky A, et al. Effects of co-occurring depression on treatment for anxiety disorders: analysis of outcomes from a large primary care effectiveness trial. J Clin Psychiatry. 2012;73(12):1509-16. doi:10.4088/JCP.12m07955
4. Moreno FA, Chong J, Dumbauld J, Humke M, Byreddy S. Use of standard Webcam and Internet equipment for telepsychiatry treatment of depression among underserved Hispanics. Psychiatr Serv. 2012;63(12):1213-7. doi:10.1176/appi.ps.201100274
5. Bombardier CH, Ehde DM, Gibbons LE, Wadhwani R, Sullivan MD, Rosenberg DE, et al. Telephone-based physical activity counseling for major depression in people with multiple sclerosis. J Consult Clin Psychol. 2013;81(1):89-99. doi:10.1037/a0031242
6. Houlihan BV, Jette A, Friedman RH, Paasche-Orlow M, Ni P, Wierbicky J, et al. A pilot study of a telehealth intervention for persons with spinal cord dysfunction. Spinal Cord. 2013;51(9):715-20. doi:10.1038/sc.2013.45
7. Chavira DA, Golinelli D, Sherbourne C, Stein MB, Sullivan G, Bystritsky A, et al. Treatment engagement and response to CBT among Latinos with anxiety disorders in primary care. J Consult Clin Psychol. 2014;82(3):392-403. doi:10.1037/a0036365
8. Christensen H, Batterham P, Mackinnon A, Griffiths KM, Kalia Hehir K, Kenardy J, et al. Prevention of generalized anxiety disorder using a web intervention, iChill: randomized controlled trial. J Med Internet Res. 2014;16(9):e199. doi:10.2196/jmir.3507
9. Pickett YR, Kennedy GJ, Freeman K, Cummings J, Woolis W. The effect of telephone-facilitated depression care on older, medically ill patients. J Behav Health Serv Res. 2014;41(1):90-6. doi:10.1007/s11414-013-9327-1
10. Gilbody S, Littlewood E, Hewitt C, Brierley G, Tharmanathan P, Araya R, et al. Computerised cognitive behaviour therapy (cCBT) as treatment for depression in primary care (REEACT trial): large scale pragmatic randomised controlled trial. BMJ. 2015;351:h5627. doi:10.1136/bmj.h5627
11. Schoene D, Valenzuela T, Toson B, Delbaere K, Severino C, Garcia J, et al. Interactive Cognitive-Motor Step Training Improves Cognitive Risk Factors of Falling in Older Adults - A Randomized Controlled Trial. PLoS One. 2015;10(12):e0145161. doi:10.1371/journal.pone.0145161
12. Hudson JL, Moss-Morris R, Game D, Carroll A, McCrone P, Hotopf M, et al. Improving distress in dialysis (iDiD): a feasibility two-arm parallel randomised controlled trial of an online cognitive behavioural therapy intervention with and without therapist-led telephone support for psychological distress in patients undergoing haemodialysis. BMJ Open. 2016;6(4):e011286. doi:10.1136/bmjopen-2016-011286
13. Moritz S, Schröder J, Klein JP, Lincoln TM, Andreou C, Fischer A, et al. Effects of online intervention for depression on mood and positive symptoms in schizophrenia. Schizophr Res. 2016;175(1-3):216-222. doi:10.1016/j.schres.2016.04.033
14. Wald I, Fruchter E, Ginat K, Stolin E, Dagan D, Bliese PD, et al. Selective prevention of combat-related post-traumatic stress disorder using attention bias modification training: a randomized controlled trial. Psychol Med. 2016;46(12):2627-36. doi:10.1017/S0033291716000945
15. Freeman D, Sheaves B, Goodwin GM, Yu LM, Nickless A, Harrison PJ, et al. The effects of improving sleep on mental health (OASIS): a randomised controlled trial with mediation analysis. Lancet Psychiatry. 2017;4(10):749-758. doi:10.1016/S2215-0366(17)30328-0
16. Hudson JL, Moss-Morris R, Norton S, Picariello F, Game D, Carroll A, et al. Tailored online cognitive behavioural therapy with or without therapist support calls to target psychological distress in adults receiving haemodialysis: A feasibility randomised controlled trial. J Psychosom Res. 2017;102:61-70. doi:10.1016/j.jpsychores.2017.09.009
17. Klein JP, Gamon C, Späth C, Berger T, Meyer B, Hohagen F, et al. Does recruitment source moderate treatment effectiveness? A subgroup analysis from the EVIDENT study, a randomised controlled trial of an internet intervention for depressive symptoms. BMJ Open. 2017;7(7):e015391. doi:10.1136/bmjopen-2016-015391
18. Klein JP, Späth C, Schröder J, Meyer B, Greiner W, Hautzinger M, et al. Time to remission from mild to moderate depressive symptoms: One year results from the EVIDENT-study, an RCT of an internet intervention for depression. Behav Res Ther. 2017;97:154-162. doi:10.1016/j.brat.2017.07.013
19. Rollman BL, Belnap BH, Mazumdar S, Abebe KZ, Karp JF, Lenze EJ, et al. Telephone-Delivered Stepped Collaborative Care for Treating Anxiety in Primary Care: A Randomized Controlled Trial. J Gen Intern Med. 2017;32(3):245-255. doi:10.1007/s11606-016-3873-1
20. Thomas Z, Novak M, Platas SGT, Gautier M, Holgin AP, Fox R, et al. Brief Mindfulness Meditation for Depression and Anxiety Symptoms in Patients Undergoing Hemodialysis: A Pilot Feasibility Study. Clin J Am Soc Nephrol. 2017;12(12):2008-2015. doi:10.2215/CJN.03900417
21. Ferrari G, Feder G, Agnew-Davies R, Bailey JE, Hollinghurst S, Howard L, et al. Psychological advocacy towards healing (PATH): A randomized controlled trial of a psychological intervention in a domestic violence service setting. PLoS One. 2018;13(11):e0205485. doi:10.1371/journal.pone.0205485
22. Gavriloff D, Sheaves B, Juss A, Espie CA, Miller CB, Kyle SD. Sham sleep feedback delivered via actigraphy biases daytime symptom reports in people with insomnia: Implications for insomnia disorder and wearable devices. J Sleep Res. 2018;27(6):e12726. doi:10.1111/jsr.12726
23. Kennard BD, Goldstein T, Foxwell AA, McMakin DL, Wolfe K, Biernesser C, et al. As Safe as Possible (ASAP): A Brief App-Supported Inpatient Intervention to Prevent Postdischarge Suicidal Behavior in Hospitalized, Suicidal Adolescents. Am J Psychiatry. 2018;175(9):864-872. doi:10.1176/appi.ajp.2018.17101151
24. Menant JC, Migliaccio AA, Sturnieks DL, Hicks C, Lo J, Ratanapongleka M, et al. Reducing the burden of dizziness in middle-aged and older people: A multifactorial, tailored, single-blind randomized controlled trial. PLoS Med. 2018;15(7):e1002620. doi:10.1371/journal.pmed.1002620
25. O'moore KA, Newby JM, Andrews G, Hunter DJ, Bennell K, Smith J, et al. Internet Cognitive-Behavioral Therapy for Depression in Older Adults With Knee Osteoarthritis: A Randomized Controlled Trial. Arthritis Care Res (Hoboken). 2018;70(1):61-70. doi:10.1002/acr.23257
26. Rollman BL, Herbeck Belnap B, Abebe KZ, Spring MB, Rotondi AJ, et al. Effectiveness of Online Collaborative Care for Treating Mood and Anxiety Disorders in Primary Care: A Randomized Clinical Trial. JAMA Psychiatry. 2018;75(1):56-64. doi: 10.1001/jamapsychiatry.2017.3379. Erratum in: JAMA Psychiatry. 2018 Jan 1;75(1):104. doi:10.1001/jamapsychiatry.2017.3977
27. Schneider BC, Schröder J, Berger T, Hohagen F, Meyer B, Späth C, et al. Corrigendum to ``Bridging the "digital divide": A comparison of use and effectiveness of an online intervention for depression between Baby Boomers and Millennials'' [Journal of Affective Disorders, 236, pp 243-251]. J Affect Disord. 2018;241:635. doi:10.1016/j.jad.2018.05.031
28. Zagorscak P, Heinrich M, Sommer D, Wagner B, Knaevelsrud C. Benefits of Individualized Feedback in Internet-Based Interventions for Depression: A Randomized Controlled Trial. Psychother Psychosom. 2018;87(1):32-45. doi:10.1159/000481515
29. Cunningham JA, Hodgins DC, Mackenzie CS, Godinho A, Schell C, Kushnir V, et al. Randomized controlled trial of an Internet intervention for problem gambling provided with or without access to an Internet intervention for co-occurring mental health distress. Internet Interv. 2019;17:100239. doi:10.1016/j.invent.2019.100239
30. Donker T, Cornelisz I, van Klaveren C, van Straten A, Carlbring P, Cuijpers P, et al. Effectiveness of Self-guided App-Based Virtual Reality Cognitive Behavior Therapy for Acrophobia: A Randomized Clinical Trial. JAMA Psychiatry. 2019;76(7):682-690. doi:10.1001/jamapsychiatry.2019.0219
31. Forand NR, Feinberg JE, Barnett JG, Strunk DR. Guided internet CBT versus "gold standard" depression treatments: An individual patient analysis. J Clin Psychol. 2019;75(4):581-593. doi:10.1002/jclp.22733
32. Gräfe V, Berger T, Hautzinger M, Hohagen F, Lutz W, Meyer B, et al. Health economic evaluation of a web-based intervention for depression: the EVIDENT-trial, a randomized controlled study. Health Econ Rev. 2019;9(1):16. doi:10.1186/s13561-019-0233-y
33. Shariful Islam SM, Chow CK, Redfern J, Kok C, Rådholm K, Stepien S, et al. Effect of text messaging on depression in patients with coronary heart disease: a substudy analysis from the TEXT ME randomised controlled trial. BMJ Open. 2019;9(2):e022637. doi:10.1136/bmjopen-2018-022637
34. Kehle-Forbes SM, Chen S, Polusny MA, Lynch KG, Koffel E, Ingram E, et al. A randomized controlled trial evaluating integrated versus phased application of evidence-based psychotherapies for military veterans with comorbid PTSD and substance use disorders. Drug Alcohol Depend. 2019;205:107647. doi:10.1016/j.drugalcdep.2019.107647
35. Miloff A, Lindner P, Dafgård P, Deak S, Garke M, Hamilton W, et al. Automated virtual reality exposure therapy for spider phobia vs. in-vivo one-session treatment: A randomized non-inferiority trial. Behav Res Ther. 2019;118:130-140. doi:10.1016/j.brat.2019.04.004
36. Niederkrotenthaler T, Till B. Effects of awareness material featuring individuals with experience of depression and suicidal thoughts on an audience with depressive symptoms: Randomized controlled trial. J Behav Ther Exp Psychiatry. 2020;66:101515. doi:10.1016/j.jbtep.2019.101515
37. Peoples AR, Garland SN, Pigeon WR, Perlis ML, Wolf JR, Heffner KL, et al. Cognitive Behavioral Therapy for Insomnia Reduces Depression in Cancer Survivors. J Clin Sleep Med. 2019;15(1):129-137. doi:10.5664/jcsm.7586
38. van der Zweerde T, van Straten A, Effting M, Kyle SD, Lancee J. Does online insomnia treatment reduce depressive symptoms? A randomized controlled trial in individuals with both insomnia and depressive symptoms. Psychol Med. 2019;49(3):501-509. doi:10.1017/S0033291718001149
39. Zalta AK, Bravo K, Valdespino-Hayden Z, Pollack MH, Burgess HJ. A placebo-controlled pilot study of a wearable morning bright light treatment for probable PTSD. Depress Anxiety. 2019;36(7):617-624. doi:10.1002/da.22897
40. Armstrong AW, Ford AR, Chambers CJ, Maverakis E, Cheng MY, Dunnick CA, et al. Comparing Online Care with In-Person Care for Patients with Psoriasis [Internet]. Washington (DC): Patient-Centered Outcomes Research Institute (PCORI); 2020. PMID: 40587629
41. Ifejika NL, Bhadane M, Cai CC, Noser EA, Grotta JC, Savitz SI. Use of a Smartphone-Based Mobile App for Weight Management in Obese Minority Stroke Survivors: Pilot Randomized Controlled Trial With Open Blinded End Point. JMIR Mhealth Uhealth. 2020;8(4):e17816. doi:10.2196/17816
42. Koranyi S, Philipp R, Quintero Garzón L, Scheffold K, Schulz-Kindermann F, Härter M, et al. Testing the Treatment Integrity of the Managing Cancer and Living Meaningfully Psychotherapeutic Intervention for Patients With Advanced Cancer. Front Psychol. 2020;11:561997. doi:10.3389/fpsyg.2020.561997
43. Kyle SD, Hurry MED, Emsley R, Marsden A, Omlin X, Juss A, et al. The effects of digital cognitive behavioral therapy for insomnia on cognitive function: a randomized controlled trial. Sleep. 2020;43(9):zsaa034. doi:10.1093/sleep/zsaa034
44. Philip KE, Lewis A, Jeffery E, Buttery S, Cave P, Cristiano D, et al. Moving singing for lung health online in response to COVID-19: experience from a randomised controlled trial. BMJ Open Respir Res. 2020;7(1):e000737. doi:10.1136/bmjresp-2020-000737
45. Rae Olmsted KL, Bartoszek M, Mulvaney S, McLean B, Turabi A, Young R, et al. Effect of Stellate Ganglion Block Treatment on Posttraumatic Stress Disorder Symptoms: A Randomized Clinical Trial. JAMA Psychiatry. 2020;77(2):130-138. doi:10.1001/jamapsychiatry.2019.3474
46. Reeves MJ, Fritz MC, Hughes AK, Woodward AT, Freddolino PP, Coursaris CK, et al. Comparing Programs to Support Stroke Survivors and Their Caregivers during the Transition Home from the Hospital or a Rehabilitation Facility—The MISTT Study [Internet]. Washington (DC): Patient-Centered Outcomes Research Institute (PCORI); 2020. PMID: 40811588
47. Russell A, Gaunt DM, Cooper K, Barton S, Horwood J, Kessler D, et al. The feasibility of low-intensity psychological therapy for depression co-occurring with autism in adults: The Autism Depression Trial (ADEPT) - a pilot randomised controlled trial. Autism. 2020;24(6):1360-1372. doi:10.1177/1362361319889272
48. Sander LB, Paganini S, Terhorst Y, Schlicker S, Lin J, Spanhel K, et al. Effectiveness of a Guided Web-Based Self-help Intervention to Prevent Depression in Patients With Persistent Back Pain: The PROD-BP Randomized Clinical Trial. JAMA Psychiatry. 2020;77(10):1001-1011. doi:10.1001/jamapsychiatry.2020.1021
49. Segal ZV, Dimidjian S, Beck A, Boggs JM, Vanderkruik R, Metcalf CA, et al. Outcomes of Online Mindfulness-Based Cognitive Therapy for Patients With Residual Depressive Symptoms: A Randomized Clinical Trial. JAMA Psychiatry. 2020;77(6):563-573. doi:10.1001/jamapsychiatry.2019.4693
50. Baumeister H, Paganini S, Sander LB, Lin J, Schlicker S, Terhorst Y, et al. Effectiveness of a Guided Internet- and Mobile-Based Intervention for Patients with Chronic Back Pain and Depression (WARD-BP): A Multicenter, Pragmatic Randomized Controlled Trial. Psychother Psychosom. 2021;90(4):255-268. doi:10.1159/000511881
51. Elbogen EB, Alsobrooks A, Battles S, Molloy K, Dennis PA, Beckham JC, et al. Mobile Neurofeedback for Pain Management in Veterans with TBI and PTSD. Pain Med. 2021;22(2):329-337. doi:10.1093/pm/pnz269
52. Fletcher S, Chondros P, Davidson S, Gunn J. Matching depression management to severity prognosis in primary care: remaining questions. Br J Gen Pract. 2024;74(744):297. doi:10.3399/bjgp24X738537
53. Klein JP, Hauer-von Mauschwitz A, Berger T, Fassbinder E, Mayer J, et al. Effectiveness and safety of the adjunctive use of an internet-based self-management intervention for borderline personality disorder in addition to care as usual: results from a randomised controlled trial. BMJ Open. 2021;11(9):e047771. doi:10.1136/bmjopen-2020-047771
54. McLean CP, Foa EB, Dondanville KA, Haddock CK, Miller ML, Rauch SAM, et al. The effects of web-prolonged exposure among military personnel and veterans with posttraumatic stress disorder. Psychol Trauma. 2021;13(6):621-631. doi:10.1037/tra0000978
55. Meyerhoff J, Haldar S, Mohr DC. The Supportive Accountability Inventory: Psychometric properties of a measure of supportive accountability in coached digital interventions. Internet Interv. 2021;25:100399. doi:10.1016/j.invent.2021.100399
56. Morriss R, Kaylor-Hughes C, Rawsthorne M, Coulson N, Simpson S, Guo B, et al. A Direct-to-Public Peer Support Program (Big White Wall) Versus Web-Based Information to Aid the Self-management of Depression and Anxiety: Results and Challenges of an Automated Randomized Controlled Trial. J Med Internet Res. 2021;23(4):e23487. doi:10.2196/23487
57. Nolan RP, Ross HJ, Farkouh ME, Huszti E, Chan S, Toma M, et al. Automated E-Counseling for Chronic Heart Failure: CHF-CePPORT Trial. Circ Heart Fail. 2021;14(1):e007073. doi:10.1161/CIRCHEARTFAILURE.120.007073
58. Nolte S, Busija L, Berger T, Meyer B, Moritz S, Rose M, et al. Do sociodemographic variables moderate effects of an internet intervention for mild to moderate depressive symptoms? An exploratory analysis of a randomised controlled trial (EVIDENT) including 1013 participants. BMJ Open. 2021;11(1):e041389. doi:10.1136/bmjopen-2020-041389
59. Owens, M., Watkins, E., Bot, M., Brouwer, I. A., Roca, M., Kohls, E., et al. Habitual behavior as a mediator between food-related behavioral activation and change in symptoms of depression in the MooDFOOD trial. Clinical Psychological Science. 2021; 9(4), 649-665. doi:10.1177/2167702620979785
60. Raevuori A, Vahlberg T, Korhonen T, Hilgert O, Aittakumpu-Hyden R, Forman-Hoffman V. A therapist-guided smartphone app for major depression in young adults: A randomized clinical trial. J Affect Disord. 2021;286:228-238. doi:10.1016/j.jad.2021.02.007
61. Scott W, Guildford BJ, Badenoch J, Driscoll E, Chilcot J, Norton S, et al. Feasibility randomized-controlled trial of online acceptance and commitment therapy for painful peripheral neuropathy in people living with HIV: The OPEN study. Eur J Pain. 2021;25(7):1493-1507. doi:10.1002/ejp.1762
62. Seal KH, Pyne JM, Manuel JK, Li Y, Koenig CJ, Zamora KA, et al. Telephone veteran peer coaching for mental health treatment engagement among rural veterans: The importance of secondary outcomes and qualitative data in a randomized controlled trial. J Rural Health. 2021;37(4):788-800. doi:10.1111/jrh.12582
63. Yang Y, Li Y, Zhang H, Xu Y, Wang B. The efficacy of computer-assisted cognitive behavioral therapy (cCBT) on psychobiological responses and perioperative outcomes in patients undergoing functional endoscopic sinus surgery: a randomized controlled trial. Perioper Med (Lond). 2021;10(1):28. doi:10.1186/s13741-021-00195-3
64. Böge K, Karnouk C, Hoell A, Tschorn M, Kamp-Becker I, Padberg F, et al. Effectiveness and cost-effectiveness for the treatment of depressive symptoms in refugees and asylum seekers: A multi-centred randomized controlled trial. Lancet Reg Health Eur. 2022;19:100413. doi:10.1016/j.lanepe.2022.100413
65. Cuijpers P, Heim E, Abi Ramia J, Burchert S, Carswell K, Cornelisz I, et al. Effects of a WHO-guided digital health intervention for depression in Syrian refugees in Lebanon: A randomized controlled trial. PLoS Med. 2022;19(6):e1004025. doi:10.1371/journal.pmed.1004025
66. Kessler RC, Furukawa TA, Kato T, Luedtke A, Petukhova M, Sadikova E, et al. An individualized treatment rule to optimize probability of remission by continuation, switching, or combining antidepressant medications after failing a first-line antidepressant in a two-stage randomized trial. Psychol Med. 2022;52(15):3371-3380. doi:10.1017/S0033291721000027
67. Langergaard A, Mathiasen K, Søndergaard J, Sørensen SS, Laursen SL, Xylander AAP, et al. Economic evaluation alongside a randomized controlled trial of blended cognitive-behavioral therapy for patients suffering from major depressive disorder. Internet Interv. 2022;28:100513. doi:10.1016/j.invent.2022.100513
68. Mathiasen K, Andersen TE, Lichtenstein MB, Ehlers LH, Riper H, Kleiboer A, et al. The Clinical Effectiveness of Blended Cognitive Behavioral Therapy Compared With Face-to-Face Cognitive Behavioral Therapy for Adult Depression: Randomized Controlled Noninferiority Trial. J Med Internet Res. 2022;24(9):e36577. doi:10.2196/36577
69. Matsumoto K, Hamatani S, Makino T, Takahashi J, Suzuki F, Ida T, et al. Guided internet-based cognitive behavioral therapy for obsessive-compulsive disorder: A multicenter randomized controlled trial in Japan. Internet Interv. 2022;28:100515. doi:10.1016/j.invent.2022
70. Oslin DW, Lynch KG, Shih MC, Ingram EP, Wray LO, Chapman SR, et al. Effect of Pharmacogenomic Testing for Drug-Gene Interactions on Medication Selection and Remission of Symptoms in Major Depressive Disorder: The PRIME Care Randomized Clinical Trial. JAMA. 2022;328(2):151-161. doi:10.1001/jama.2022.9805
71. Paul M, Bullock K, Bailenson J. Virtual Reality Behavioral Activation for Adults With Major Depressive Disorder: Feasibility Randomized Controlled Trial. JMIR Ment Health. 2022;9(5):e35526. doi:10.2196/35526
72. Zheng F, Zheng Y, Liu S, Yang J, Xiao W, Xiao W, et al. The Effect of M-Health-Based Core Stability Exercise Combined with Self-Compassion Training for Patients with Nonspecific Chronic Low Back Pain: A Randomized Controlled Pilot Study. Pain Ther. 2022;11(2):511-528. doi:10.1007/s40122-022-00358-0
73. Benjet C, Zainal NH, Albor Y, Alvis-Barranco L, Carrasco-Tapias N, Contreras-Ibáñez CC, et al. A Precision Treatment Model for Internet-Delivered Cognitive Behavioral Therapy for Anxiety and Depression Among University Students: A Secondary Analysis of a Randomized Clinical Trial. JAMA Psychiatry. 2023;80(8):768-777. doi:10.1001/jamapsychiatry.2023.1675
74. Connell AM, Stormshak EA. Evaluating the Efficacy of the Family Check-Up Online to Improve Parent Mental Health and Family Functioning in Response to the COVID-19 Pandemic: A Randomized Clinical Trial. J Prev (2022). 2023;44(3):341-357. doi:10.1007/s10935-023-00727-1
75. Grobelski J, Wilsmann-Theis D, Karakostas P, Behning C, Brossart P, Schäfer VS. Prospective double-blind study on the value of musculoskeletal ultrasound by dermatologists as a screening instrument for psoriatic arthritis. Rheumatology (Oxford). 2023;62(8):2724-2731. doi:10.1093/rheumatology/keac702
76. Hellrigel-Holderbaum M, Romanczuk-Seiferth N, Glos M, Fietze I. Effects of mindfulness meditation and Acceptance and commitment therapy in patients with obstructive sleep apnea with residual excessive sleepiness: A randomized controlled pilot study. Sleep Med. 2023;106:33-41. doi:10.1016/j.sleep.2023.03.022
77. Ishrat Husain M, Rodie DJ, Perivolaris A, Sanches M, Crawford A, Fitzgibbon KP, et al. A Collaborative-Care Telephone-Based Intervention for Depression, Anxiety, and at-Risk Drinking in Primary Care: The PARTNERs Randomized Clinical Trial. Can J Psychiatry. 2023;68(10):732-744. doi:10.1177/07067437231156243
78. Jelinek L, Schröder J, Bücker L, Miegel F, Baumeister A, Moritz S, et al. Improving adherence to unguided Internet-based therapy for obsessive-compulsive disorder: A randomized controlled trial. J Affect Disord. 2023;338:472-481. doi:10.1016/j.jad.2023.06.038
79. Kerber A, Beintner I, Burchert S, Knaevelsrud C. Effects of a Self-Guided Transdiagnostic Smartphone App on Patient Empowerment and Mental Health: Randomized Controlled Trial. JMIR Ment Health. 2023;10:e45068. doi:10.2196/45068
80. Lester EG, Wang KE, Blakeley JO, Vranceanu AM. Occurrence and Severity of Suicidal Ideation in Adults With Neurofibromatosis Participating in a Mind-Body RCT. Cogn Behav Neurol. 2023;36(1):19-27. doi:10.1097/WNN.0000000000000332
81. Lu Y, Li Y, Huang Y, Zhang X, Wang J, Wu L, et al. Effects and Mechanisms of a Web- and Mobile-Based Acceptance and Commitment Therapy Intervention for Anxiety and Depression Symptoms in Nurses: Fully Decentralized Randomized Controlled Trial. J Med Internet Res. 2023;25:e51549. doi:10.2196/51549
82. McGillivray L, Keng-Meng Hui N, Wong QJJ, Han J, Qian J, Torok M. The Effectiveness of a Smartphone Intervention Targeting Suicidal Ideation in Young Adults: Randomized Controlled Trial Examining the Influence of Loneliness. JMIR Ment Health. 2023;10:e44862. doi:10.2196/44862
83. Mide M, Mattiasson J, Norlin D, Sehlin H, Rasmusson J, Ljung S, et al. Internet-delivered therapist-assisted cognitive behavioral therapy for gambling disorder: a randomized controlled trial. Front Psychiatry. 2023;14:1243826. doi:10.3389/fpsyt.2023.1243826
84. Moritz S, Penney D, Missmann F, Weidinger S, Schmotz S. Self-Help Habit Replacement in Individuals With Body-Focused Repetitive Behaviors: A Proof-of-Concept Randomized Clinical Trial. JAMA Dermatol. 2023;159(9):992-995. doi:10.1001/jamadermatol.2023.2167
85. Morriss R, Patel S, Boutry C, Patel P, Guo B, Briley PM, et al. Clinical effectiveness of active Alpha-Stim AID versus sham Alpha-Stim AID in major depression in primary care in England (Alpha-Stim-D): a multicentre, parallel group, double-blind, randomised controlled trial. Lancet Psychiatry. 2023;10(3):172-183. doi:10.1016/S2215-0366(23)00007-X
86. Poole N, Cope S, Vanzan S, Duffus A, Mantovani N, Smith J, et al. Feasibility randomised controlled trial of online group Acceptance and Commitment Therapy for Functional Cognitive Disorder (ACT4FCD). BMJ Open. 2023;13(5):e072366. doi:10.1136/bmjopen-2023-072366
87. Stewart JC, Patel JS, Polanka BM, Gao S, Nurnberger JI, MacDonald KL, et al. Effect of modernized collaborative care for depression on depressive symptoms and cardiovascular disease risk biomarkers: eIMPACT randomized controlled trial. Brain Behav Immun. 2023;112:18-28. doi:10.1016/j.bbi.2023.05.007
88. Strauss C, Bibby-Jones AM, Jones F, Byford S, Heslin M, Parry G, et al. Clinical Effectiveness and Cost-Effectiveness of Supported Mindfulness-Based Cognitive Therapy Self-help Compared With Supported Cognitive Behavioral Therapy Self-help for Adults Experiencing Depression: The Low-Intensity Guided Help Through Mindfulness (LIGHTMind) Randomized Clinical Trial. JAMA Psychiatry. 2023;80(5):415-424. doi:10.1001/jamapsychiatry.2023.0222
89. Werner-Seidler A, Li SH, Spanos S, Johnston L, O'Dea B, Torok M, et al. The effects of a sleep-focused smartphone application on insomnia and depressive symptoms: a randomised controlled trial and mediation analysis. J Child Psychol Psychiatry. 2023;64(9):1324-1335. doi:10.1111/jcpp.13795
90. Bauer JF, Gerczuk M, Schindler-Gmelch L, Amiriparian S, Ebert DD, Krajewski J, et al. Validation of Machine Learning-Based Assessment of Major Depressive Disorder from Paralinguistic Speech Characteristics in Routine Care. Depress Anxiety. 2024;2024:9667377. doi:10.1155/2024/9667377
91. Daros AR, Guimond TH, Yager C, Palermo EH, Wilks CR, Quilty LC. Feasibility, Acceptability, and Potential Efficacy of a Self-Guided Internet-Delivered Dialectical Behavior Therapy Intervention for Substance Use Disorders: Randomized Controlled Trial. JMIR Ment Health. 2024;11:e50399. doi:10.2196/50399
92. Gata A, Raduly L, Budișan L, Bajcsi A, Ursu TM, Chira C, et al. Machine Learning Model Predicts Postoperative Outcomes in Chronic Rhinosinusitis With Nasal Polyps. Clin Otolaryngol. 2024;49(6):776-784. doi:10.1111/coa.14208
93. Gitaari M, Mikolic A, Panenka WJ, Silverberg ND. Diagnostic Accuracy of Mental Health Screening Tools After Mild Traumatic Brain Injury. JAMA Netw Open. 2024;7(7):e2424076. doi:10.1001/jamanetworkopen.2024.24076
94. Guo W, Nazari N, Sadeghi M. Cognitive-behavioral treatment for insomnia and mindfulness-based stress reduction in nurses with insomnia: a non-inferiority internet delivered randomized controlled trial. PeerJ. 2024;12:e17491. doi:10.7717/peerj.17491
95. Jagayat JK, Kumar A, Shao Y, Pannu A, Patel C, Shirazi A, et al. Incorporating a Stepped Care Approach Into Internet-Based Cognitive Behavioral Therapy for Depression: Randomized Controlled Trial. JMIR Ment Health. 2024;11:e51704. doi:10.2196/51704
96. Kim Y, Kim M, Kim J, Song TJ. Smartphone-Based Speech Therapy for Poststroke Dysarthria: Pilot Randomized Controlled Trial Evaluating Efficacy and Feasibility. J Med Internet Res. 2024;26:e56417. doi:10.2196/56417
97. Kleinau E, Lamba T, Jaskiewicz W, Gorentz K, Hungerbuehler I, Rahimi D, et al. Effectiveness of a chatbot in improving the mental wellbeing of health workers in Malawi during the COVID-19 pandemic: A randomized, controlled trial. PLoS One. 2024;19(5):e0303370. doi:10.1371/journal.pone.0303370
98. Lee C, Mohebbi M, O'Callaghan E, Winsberg M. Large Language Models Versus Expert Clinicians in Crisis Prediction Among Telemental Health Patients: Comparative Study. JMIR Ment Health. 2024;11:e58129. doi:10.2196/58129
99. Li Q, Wang H, Zhong B, Zhang T, Wang Z, Tao P, et al. Conventional versus high-voltage, long-term pulse Radiofrequency of ganglion impar in perineal pain with advanced rectal cancer: a Randomized, double-blind controlled trial. BMC Anesthesiol. 2024;24(1):327. doi:10.1186/s12871-024-02717-0
100. Liang R, Tang L, Li L, Zhao N, Yu X, Li J, et al. The effect of pressing needle therapy on depression, anxiety, and sleep for patients in convalescence from COVID-19. Front Neurol. 2024;15:1481557. doi:10.3389/fneur.2024.1481557
101. Maulik PK, Daniel M, Devarapalli S, Kallakuri S, Kaur A, Ghosh A, et al. Mental Health Care Support in Rural India: A Cluster Randomized Clinical Trial. JAMA Psychiatry. 2024;81(11):1061-1070. doi:10.1001/jamapsychiatry.2024.2305
102. O'Neil A, Perez J, Young LM, John T, Turner M, Saunders D, et al. Clinical and cost-effectiveness of remote-delivered, online lifestyle therapy versus psychotherapy for reducing depression: results from the CALM non-inferiority, randomised trial. Lancet Reg Health West Pac. 2024;49:101142. doi:10.1016/j.lanwpc.2024.101142
103. Philip KEJ, Buttery SC, Bowen S, Lewis A, Jeffery E, Alghamdi SM, et al. Singing for lung health in COPD: a multicentre randomised controlled trial of online delivery. BMJ Open Respir Res. 2024;11(1):e002365. doi:10.1136/bmjresp-2024-002365
104. Picariello F, Hulme K, Seaton N, Hudson JL, Norton S, Wroe A, et al. A randomized controlled trial of a digital cognitive-behavioral therapy program (COMPASS) for managing depression and anxiety related to living with a long-term physical health condition. Psychol Med. 2024;54(8):1796-1809. doi:10.1017/S0033291723003756
105. Safiah MH, Kalalib Al Ashabi K, Alchallah MO, Alolabi H, Ataya S, et al. Symptoms of acute stress, anxiety, and depression following Türkiye-Syria earthquake among Syrian population: national survey during the acute phase. Eur J Psychotraumatol. 2024;15(1):2433836. doi:10.1080/20008066.2024.2433836
106. Shi S, Cui S, Yao Y, Ge M, Yang M, Sheng X, et al. Smartphone video games improve cognitive function in patients with chronic schizophrenia: a randomized controlled trial. Eur Arch Psychiatry Clin Neurosci. 2024;274(4):929-939. doi:10.1007/s00406-023-01660-4
107. Song H, Edwards C 2nd, Curto R, Perez A, Cruess C, Schell A, et al. Does Epidural Corticosteroid Application During Spinal Surgery Reduce Postoperative Pain?: An Adjunct to Multimodal Analgesia. Clin Spine Surg. 2024;37(8):E354-E363. doi:10.1097/BSD.0000000000001586
108. Woolf C, Kaplan L, Norrie LM, Burke D, Cunich M, Mowszowski L, et al. A feasibility, randomised controlled trial of Club Connect: a group-based healthy brain ageing cognitive training program for older adults with major depression within an older people's mental health service. BMC Psychiatry. 2024;24(1):208. doi:10.1186/s12888-023-05391-7
109. Xiang X, Kayser J, Turner S, Ash S, Himle JA. Layperson-Supported, Web-Delivered Cognitive Behavioral Therapy for Depression in Older Adults: Randomized Controlled Trial. J Med Internet Res. 2024;26:e53001. doi:10.2196/53001
110. Xu W, Zhao N, Li W, Qiu L, Luo X, Lin Y, et al. Effects of repetitive transcranial magnetic stimulation on fear of cancer recurrence and its underlying neuromechanism. Contemp Clin Trials Commun. 2024;39:101299. doi:10.1016/j.conctc.2024.101299
111. Yatziv SL, Pedrelli P, Baror S, DeCaro SA, Shachar N, Sofer B, et al. Facilitating Thought Progression to Reduce Depressive Symptoms: Randomized Controlled Trial. J Med Internet Res. 2024;26:e56201. doi:10.2196/56201
112. Jia Y, Kunaviktikul W, Panuthai S, Chaiard J. Effects of Trajectory-based Support Via WeChat in Reducing Burden and Depression among Carers of People after Stroke: A Randomized Controlled Trial. PRIJNR. 2024;28(4):838-54. doi:10.60099/prijnr.2024.268856
113. Aktürk T, Agargün MY, Özdemir S, Dalmizrak E, Güntekin B. Home-use transcranial direct current stimulation (tDCS) as a safe, effective, and feasible application in depression: A randomized, single-blind, placebo-controlled trial. Journal of Affective Disorders Reports. 2025;20:100902. doi:10.1016/j.jadr.2025.100902
114. Assmann N, Jacob G, Schaich A, Berger T, Zindler T, Betz L, et al. A digital therapeutic for people with borderline personality disorder in Germany (EPADIP-BPD): a pragmatic, assessor-blind, parallel-group, randomised controlled trial. Lancet Psychiatry. 2025;12(5):366-376. doi:10.1016/S2215-0366(25)00063-X
115. Cheng C, Jia M, Peng X, Sun Y, Jiao Y, Zhang M, et al. Different regulative effects of high- and low-frequency external trigeminal nerve stimulation (eTNS) on sleep activity: Preliminary study. Sleep Med. 2025;125:136-145. doi:10.1016/j.sleep.2024.11.007
116. Fleischhauer T, Sander N, Feisst M, Awounvo S, Weller L, Poss-Doering R, et al, Müller-Bühl U, Szecsenyi J, Senft JD. Treating Venous Leg Ulcers in Primary Care: The Cluster-Randomized Ulcus Cruris Care Trial. Dtsch Arztebl Int. 2026;123(1):9-14. doi:10.3238/arztebl.m2025.0207
117. Hentati A, Hentati Isacsson N, Rosén A, Jernelöv S, Kaldo V et al. Effects of intervention design on engagement and outcomes in digital self-help for insomnia - factorial RCT. NPJ Digit Med. 2025;8(1):416. doi:10.1038/s41746-025-01839-0
118. Herrero R, Franke M, Görlich D, Garcia-Palacios A, Baños R, Jacobi C, et al. Efficacy of the internet-based intervention "Cultivating our resilience" (CORE) for improving resilience and coping strategies in university students: A randomized controlled trial. Internet Interv. 2025;39:100811. doi:10.1016/j.invent.2025.100811
119. Kern D, Ljótsson B, Lönndahl L, Hedman-Lagerlöf E, Molander O, Liliequist B, et al. Self-Guided vs Clinician-Guided Online Cognitive Behavioral Therapy for Atopic Dermatitis: A Randomized Clinical Trial. JAMA Dermatol. 2025;161(2):183-190. doi:10.1001/jamadermatol.2024.5044
120. Kusumadewi AF, Marchira CR, Widyandana D, Wirasto RT. Randomized clinical trial comparing the effects of an asynchronous mobile application to guided brief cognitive behavioral therapy for managing anxiety among medical students. Trends Psychiatry Psychother. 2025;47:e20230713. doi:10.47626/2237-6089-2023-0713
121. Lee M, Jang S, Shin HK, Choi SW, Kim HT, Oh J, et al. Virtual Reality-Based Cognitive Behavior Therapy for Major Depressive Disorder: An Alternative to Pharmacotherapy for Reducing Suicidality. Yonsei Med J. 2025;66(1):25-36. doi:10.3349/ymj.2024.0002
122. Leung LB, Brayton CE, Hovsepian S, Karakashian MA, Chu K, Jackson NJ, et al. Adapting depression collaborative care models to increase uptake of computerized cognitive behavioral therapy at the VA: A pilot randomized controlled trial. Gen Hosp Psychiatry. 2025;96:223-233. doi:10.1016/j.genhosppsych.2025.07.014
123. Morriss R, Webster L, Ingram L, Abdelghani M, Anton A, Barber S, et al. Connectivity guided intermittent theta burst stimulation versus repetitive transcranial magnetic stimulation in moderately severe treatment resistant depression. Southampton (UK): National Institute for Health and Care Research; 2025. PMID: 39999247
124. Nilkantham S, Singh A, Majumdar V, K N H, Atmakur S. Enhancing Quality of Life in Patients With Hypothyroidism Using a Scientific Yoga Module: Randomized Controlled Trial. J Med Internet Res. 2025;27:e54078. doi:10.2196/54078
125. O'Driscoll C, O'Reilly S, Jaremba AJ, Nolte T, Shaikh M. Smartphone-Based Mindfulness and Mentalization Ecological Momentary Interventions for Common Mental Health Problems: Pilot Randomized Controlled Trial. JMIR Ment Health. 2025;12:e79296. doi:10.2196/79296
126. Pieh C, Humer E, Hoenigl A, Schwab J, Mayerhofer D, Dale R, et al. Smartphone screen time reduction improves mental health: a randomized controlled trial. BMC Med. 2025;23(1):107. doi:10.1186/s12916-025-03944-z
127. Poole N, Cope S, Vanzan S, Duffus A, Williams T, Mantovani N, et al. Randomised controlled feasibility trial of online group acceptance and commitment therapy for functional cognitive disorder. BJPsych Open. 2025;11(3):e91. doi:10.1192/bjo.2025.33
128. Prott LS, Kaldenhoven V, Hugger A, Langner R, Gierthmuehlen PC, Gierthmuehlen M. Transcutaneous Auricular Vagus Nerve Stimulation for the Treatment of Myoarthropathic Symptoms Associated With Temporomandibular Disorders-A Pilot Randomised Controlled Trial. J Oral Rehabil. 2025;52(12):2351-2360. doi:10.1111/joor.70039
129. Ravitz P, Heeney N, Lawson A, McAnanama E, Pain C, Kiss A, et al. Scaling up interpersonal psychotherapy training: A pilot randomized controlled trial of digital asynchronous self-directed vs synchronous group workshop training. J Affect Disord Rep. 2025;19:100863. doi:10.1016/j.jadr.2024.100863
130. Rotstein NM, Cohen ZD, Welborn A, Zbozinek TD, Akre S, Jones KG, et al. Investigating low intensity focused ultrasound pulsation in anhedonic depression-A randomized controlled trial. Front Hum Neurosci. 2025;19:1478534. doi:10.3389/fnhum.2025.1478534
131. Schmidt M, Schoenenberg K, Engelkamp JE, Staufenbiel T, Martin A, Ebert DD, et al. Efficacy of an internet-based, therapist-guided cognitive behavioral therapy intervention for adolescents and young adults with body dysmorphic disorder: a randomized controlled trial. BMC Psychiatry. 2025;25(1):374. doi:10.1186/s12888-025-06797-1
132. Simpson LA, Barclay R, Bayley MT, Brasher PMA, Dukelow SP, MacIntosh BJ, et al. A Randomized Control Trial of a Virtually Delivered Program for Increasing Upper Limb Activity After Stroke. Neurorehabil Neural Repair. 2025;39(3):179-189. doi:10.1177/15459683241303702
133. Stadler M, Zaremba N, Harrison A, Brown J, Pillay D, Allan J, et al. Safety of a co-designed cognitive behavioural therapy intervention for people with type 1 diabetes and eating disorders (STEADY): a feasibility randomised controlled trial. Lancet Reg Health Eur. 2025;50:101205. doi:10.1016/j.lanepe.2024.101205
134. Taguchi K, Miyoshi M, Seki Y, Baba S, Shimizu E. Effectiveness of Cognitive Behavioral Therapy Provided Through a Web Application for Subthreshold Depression, Subthreshold Insomnia, and Subthreshold Panic: Open-Labeled 6-Arm Randomized Clinical Trial Pilot Study. JMIR Form Res. 2025;9:e63139. doi:10.2196/63139
135. Tong ACY, Wong KTY, Chung WWT, Mak WWS. Effectiveness of Topic-Based Chatbots on Mental Health Self-Care and Mental Well-Being: Randomized Controlled Trial. J Med Internet Res. 2025;27:e70436. doi:10.2196/70436
136. Tulbure BT, Dudău DP, Marian Ș, Watkins E. An Internet-Delivered Rumination-Focused CBT Intervention for Adults With Depression and Anxiety: A Randomized Controlled Trial. Behav Ther. 2025;56(4):785-798. doi:10.1016/j.beth.2024.12.004
137. Vall-Roqué H, Ramos-García V, Rivero-Santana A, Torres-Castaño A, Cifuentes P, Koatz D, et al. A virtual community of practice to empower patients with recent ischemic heart disease: A randomized controlled trial. Health Psychol. 2025;44(6):630-642. doi:10.1037/hea0001439
138. Yan F, Xiao L, Huang C, Tang S, Li L. Effectiveness of a nurse-led hybrid self-management program for community-dwelling older people with urinary incontinence: A randomized controlled trial. Int J Nurs Stud. 2025;170:105157. doi:10.1016/j.ijnurstu.2025.105157
139. Yang JH, Keun Park CH, Rhee SJ, Kang DH, Lee SY, Yang CM, et al. Effectiveness of internet-based cognitive behavioral therapy in reducing suicidality among high-risk patients with depression: A multicenter randomized controlled trial in Korea. J Psychiatr Res. 2025;181:640-646. doi:10.1016/j.jpsychires.2024.12.034
140. Zhang S, Wang Y, Wu J, Ma C, Meng X. Effectiveness of Smartwatch Device on Adherence to Home-Based Cardiac Rehabilitation in Patients With Coronary Heart Disease: Randomized Controlled Trial. JMIR Mhealth Uhealth. 2025;13:e70848. doi:10.2196/70848
141. Saeed S, Wang H, Kong L, Geng Y, Zhang J, Pan Y, et al. Machine learning-enhanced mapping of suicide risk in Bipolar Disorder: A multi-modal analysis. J Affect Disord. 2026;392:120183. doi:10.1016/j.jad.2025.120183
