# Supplementary material for: Magnitude and Moderators of Depressive Symptom Improvement in Digital Placebo Arms: Systematic Review and Meta-Analysis
Source: J Med Internet Res. 2026 Oct 9;28:e100426. doi: 10.2196/100426 (PMC13695001; doi:10.2196/100426)
Supplement: Supplementary file 3 [file jmir_v28i1e100426_app3.docx]

Multimedia Appendix 3. Characteristics of Trials Included in this Study

| Characteristics | N (%) | Characteristics | N (%) |
| --- | --- | --- | --- |
| Publication year  　- 2015  　2016 - 2020  　2021 - | 3 (10.0)  3 (10.0)  24 (80.0) | Total number of participants  　- 100  　101 - 500  　501 - 1000  　1001 - | 6 (20.0)  14 (46.7)  8 (26.7)  2 (6.7) |
| Target population  　Non-patients  　Other diseases  　Primary psychiatric disorder | 17 (56.7)  4 (13.3)  9 (30.0) | Blindness  　Double^e^  　Single-I^f^  　Single-P^g^ | 9 (30.0)  9 (30.0)  12 (40.0) |
| Mean age  　- 20  　20 - 40  　40 - 60  　60 –  　Not specified | 3 (10.0)  13 (43.3)  8 (26.7)  4 (13.3)  2 (6.7) | Treatment period (day)  　1 - 30  　31 - 60  　61 - | 10 (33.3)  13 (43.3)  7 (23.3) |
| Placebo delivery type  　Computer  　Mobile  　Wearable  　Web  　Web/Mobile | 1 (3.3)  13 (43.3)  1 (3.3)  9 (30.0)  6 (20.0) | Number of groups  　2  　3 | 24 (80.0)  6 (20.0) |
| Placebo approach  　Less  　Replaced  　Removed  　Unrelated | 1 (3.3)  17 (56.7)  7 (23.3)  5 (16.7) | Region  　APAC^a^  　EMEA^b^  　LATAM^c^  　NA^d^ | 13 (43.3)  11 (36.7)  2 (6.7)  4 (13.3) |
| Endpoint type  　Primary  　Secondary | 15 (50.0)  15 (50.0) | Risk of bias  　Low  　Some concerns  　High | 11 (36.7)  15 (50.0)  4 (13.3) |

^a^APAC: Asia-Pacific

^b^EMEA: Europe, Middle East and Africa

^c^LATAM: Latin America

^d^ NA: North America

^e^Double: Both participants and investigators are blinded

^e^Single-I: Only investigators assessors are blinded

^f^Single-P: Only participants are blinded
