# Supplementary material for: Magnitude and Moderators of Depressive Symptom Improvement in Digital Placebo Arms: Systematic Review and Meta-Analysis
Source: J Med Internet Res. 2026 Oct 9;28:e100426. doi: 10.2196/100426 (PMC13695001; doi:10.2196/100426)
Supplement: Supplementary file 4 [file jmir_v28i1e100426_app4.docx]

Multimedia Appendix 4. Funnel Plot
